# Turing-pattern model of scaffolding proteins that establish spatial asymmetry during the cell cycle of *Caulobacter crescentus*

**DOI:** 10.1016/j.isci.2023.106513

**Published:** 2023-03-29

**Authors:** Chunrui Xu, John J. Tyson, Yang Cao

**Affiliations:** 1 School of Life Sciences, Zhengzhou University, Zhengzhou 450000 Henan, China; 2 Program in Genetics, Bioinformatics, and Computational Biology, Virginia Tech, Blacksburg 24061 VA, USA; 3 Department of Biological Sciences, Virginia Tech, Blacksburg 24061 VA, USA; 4 Department of Computer Science, Virginia Tech, Blacksburg 24061 VA, USA

**Keywords:** Microbiology, Cell biology, Protein folding

## Abstract

The crescent-shaped bacterium *Caulobacter crescentus* divides asymmetrically into a sessile (stalked) cell and a motile (flagellated) cell. This dimorphic cell division cycle is driven by the asymmetric appearance of scaffolding proteins at the cell’s stalk and flagellum poles. The scaffolding proteins recruit enzyme complexes that phosphorylate and degrade a master transcription factor, CtrA, and the abundance and phosphorylation state of CtrA control the onset of DNA synthesis and the differentiation of stalked and flagellated cell types. In this study, we use a Turing-pattern mechanism to simulate the spatiotemporal dynamics of scaffolding proteins in *Caulobacter* and how they influence the abundance and intracellular distribution of CtrA∼P. Our mathematical model captures crucial features of wild-type and mutant strains and predicts the distributions of CtrA∼P and signaling proteins in mutant strains. Our model accounts for *Caulobacter* polar morphogenesis and shows how spatial localization and phosphosignaling cooperate to establish asymmetry during the cell cycle.

## Highlights

• *Caulobacter* cells divide asymmetrically into a stalked cell and a flagellated cell
• Asymmetry is built and maintained by protein polymerization at the cell’s poles
• Polymerization is driven by a Turing mechanism of spontaneous symmetry breaking
• Mathematical model accounts for protein localization in wild-type and mutant cells

## Graphical abstract

**Figure:**
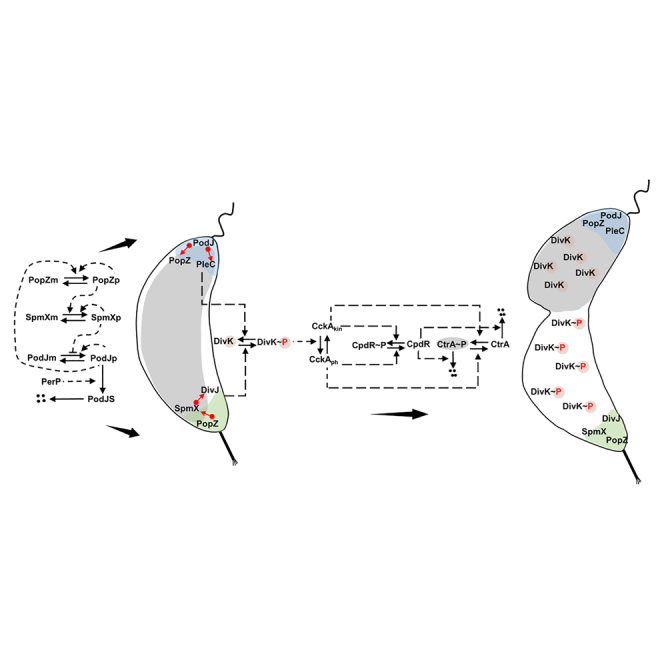

## Introduction

**Figure 1:**
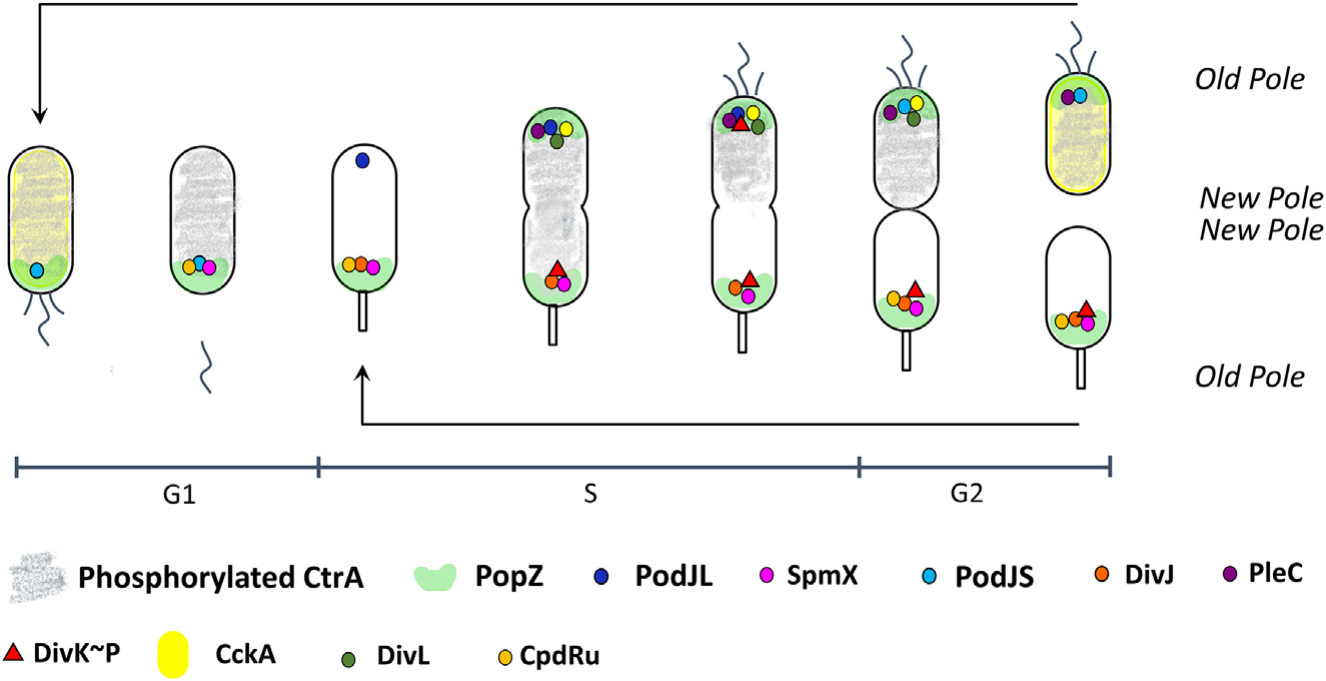
Dynamic localization of key proteins over the cell cycle of *C*. *crescentus* CtrA∼P develops an asymmetric spatial distribution during the cell cycle (gray). The three scaffolding proteins, PopZ, PodJ, and SpmX, interact with each other and recruit (directly or indirectly) client proteins at specific poles, including DivJ,^10^ PleC,^11^ DivK,^10^ CckA,^12^ DivL,^12^ and CpdR.^13^ CckA (fluorescent yellow) is uniformly distributed throughout the cell in the swarmer cell and localized at the new pole during the predivisional stage. Some *Caulobacter* predivisional cells may also have old polar localized CckA (not shown).^14^

In prokaryotic cells, asymmetric protein distributions contribute to diverse physiological processes including morphogenesis, stress response, and signal transduction.^1^^,^^2^ A model organism for studying bacterial cell asymmetry is the oligotrophic aquatic bacterium, *Caulobacter crescentus*, in which at least 10% of proteins are non-uniformly distributed across a cell.^2^^,^^3^ *Caulobacter* cells undergo a dimorphic division cycle, regulated by asymmetrically distributed proteins such as CtrA, a potent inhibitor of DNA replication.^4^^,^^5^ Cell division produces two distinct progeny: a motile swarmer cell with high levels of phosphorylated CtrA (CtrA∼P) and a sessile stalked cell with low levels of CtrA∼P^6^ (Figure 1). As phosphorylated CtrA inhibits the initiation of DNA replication by binding to the chromosome origin of replication (*Cori*), the swarmer cell is blocked from DNA replication and cell division. To reproduce, the swarmer cell must eliminate CtrA∼P, by degradation and/or dephosphorylation, a process that occurs during the swarmer-to-stalked (sw-to-st) transition. During this transition, the swarmer cell sheds its flagellum and makes a stalk. The stalk cell commences DNA replication and begins to generate a flagellum at the opposite pole. The predivisional cell, with a stalk at the “old” pole, a flagellum at the “new” pole, and a partially or fully replicated chromosome in the middle, exhibits dynamic localization of key proteins at the old and new poles (Figure 1). At cell division, the motile swarmer cell separates from the sessile stalk cell. The stalked cell recommences the cell division cycle immediately after cell division.^7^^,^^8^^,^^9^

In addition to the changing asymmetric distributions of signaling proteins, cell cycle progression in *Caulobacter* is also controlled by temporal patterns of gene expression through the actions of three transcription factors (CtrA, GcrA, and DnaA) and a DNA methylase (CcrM).^15^^,^^16^ GcrA and DnaA (unlike CtrA) promote the initiation of DNA replication, which converts fully methylated DNA sequences to hemi-methylated conditions. Later in the cycle, these sequences are returned to the fully methylated state by CcrM. The methylation state of genes affects their transcriptional activities.

The temporal dynamics of this complex network of interacting transcription factors and DNA methylation has been modeled mathematically by Li et al.^17^^,^^18^ and Shen et al.,^19^ who have reproduced *in silico* the time courses of key regulatory proteins in wild-type (WT) and mutant cells and predicted the phenotypes of novel mutants. Murray et al.^20^ studied a simpler model involving just GcrA, CtrA, and CcrM. Using a CtrA-centric model, Weston et al.^21^ suggested that unphosphorylated CtrA competes with phosphorylated CtrA for binding to *Cori*. These “temporal” models are helpful in understanding how the underlying molecular control system governs the sequence of events in the *Caulobacter* cell cycle, but they cannot account for the spatial localization of regulatory proteins, which is so fundamental to asymmetric cell division in *Caulobacter*.

**Figure 2:**
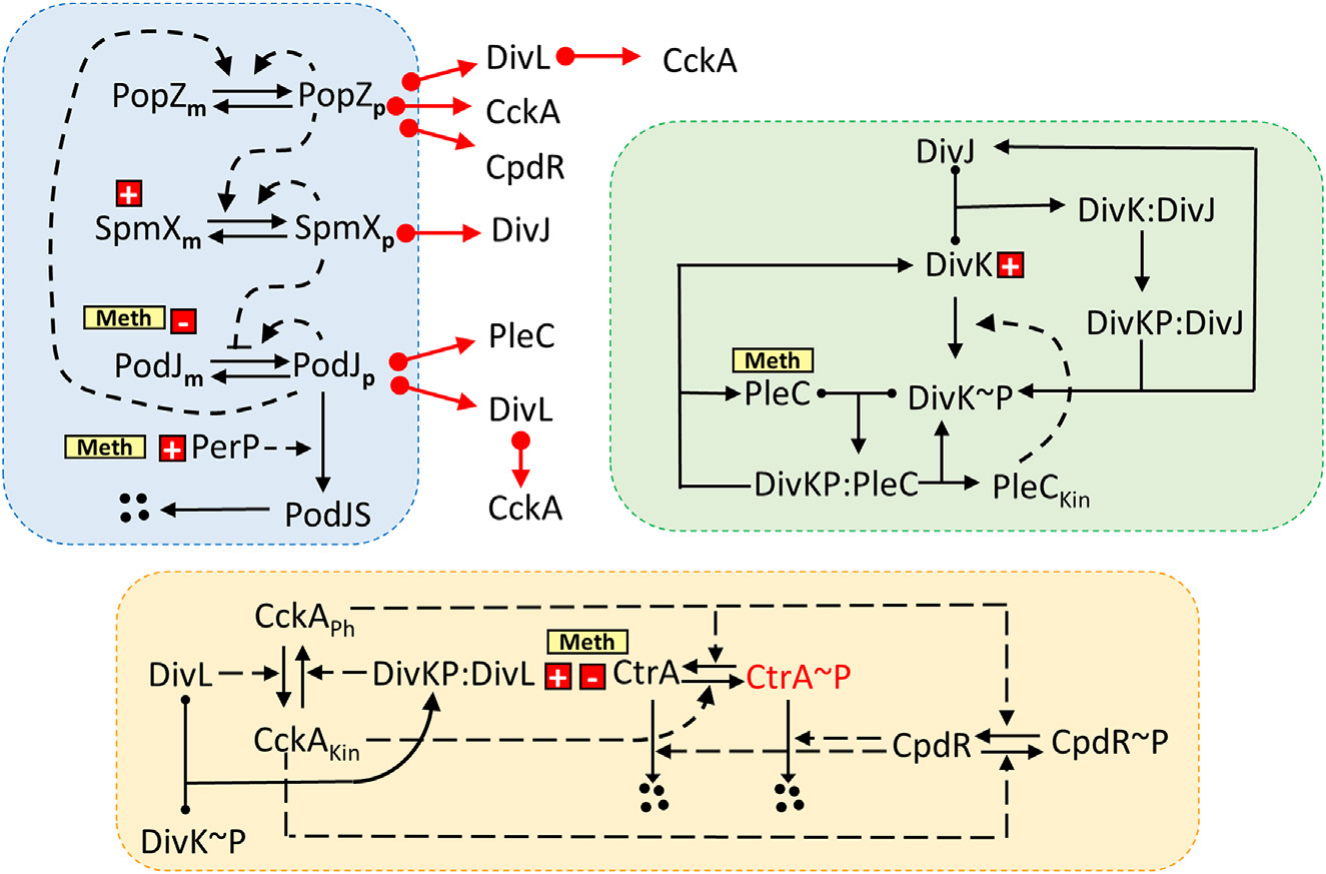
Schematic diagram of the spatiotemporal regulatory network in *Caulobacter* Red solid lines with an arrow at one end and a circle at the other end indicate localization effects of scaffolding proteins, pointing from the scaffolding protein to the client protein. Black dashed lines indicate enzymatic activities. “Meth” indicates methylation-controlled transcription. The red squares with minus or plus represent CtrA-regulated inhibition or activation of transcriptions. Four black spots represent products of degradation. The trident with solid circles at two arms and an arrow at the third arm indicates binding of two species. For example, DivJ binds to DivK to form the complex DivK:DivJ. PodJ = long form of PodJ; PodJS = short form of PodJ. Other abbreviations: m = monomer, p = polymer, Kin = kinase, Ph = phosphatase.

In this paper, because our focus is on spatial aspects of the control system, we ignore (for now) the roles of GcrA and DnaA, simplify the modeling of CcrM, and focus on the spatiotemporal changes in CtrA activity. The abundance of active CtrA is determined by three pathways: synthesis, proteolysis, and phosphosignaling. The *ctrA* gene has two promoters: the weaker promoter (P1) is inhibited by CtrA∼P and the stronger promoter (P2) is activated by CtrA∼P. The degradation and phosphorylation of CtrA are regulated by two phosphotransfer modules: DivL-CckA-CtrA and DivJ/PleC-DivK (yellow and green boxes in Figure 2, respectively). CckA and PleC are bifunctional histidine kinases working as either a kinase or phosphatase for their response regulators, CtrA and DivK, respectively.^22^^,^^23^^,^^24^^,^^25^ CckA mediates the phosphorylation/dephosphorylation of both CtrA and CpdR. Unphosphorylated CpdR is an essential component of the ClpXP protease complex specifically responsible for the degradation of CtrA.^14^ Thus, CckA regulates the activity of CtrA through both phosphotransfer and proteolysis.

Although CckA level remains constant throughout the cell cycle, its subcellular localization varies.^26^ Time-lapse microscopy indicates that CckA has no preferential localization in a swarmer cell and accumulates at the flagellated pole (new pole) during stalked and predivisional stages (Figure 1, fluorescent yellow). Around 30% of WT cells have strong old-pole accumulation of CckA during the stalked stage, which suggests that the old pole may serve as a depot for surplus CckA and that old-polar localization of CckA is optional for normal cell cycle progression.^14^^,^^24^

CckA’s switch between kinase and phosphatase is allosterically mediated by DivL and DivK∼P.^27^ DivL stimulates the kinase activity of CckA, whereas CckA binding with DivL:DivK∼P stimulates its phosphatase activity.^3^^,^^27^^,^^28^ Therefore, the interaction between DivL and DivK∼P links the two phosphotransfer modules. In addition, DivL is required for new-polar localization of CckA.^27^^,^^29^ PopZ is another binding partner of CckA, which may contribute to its localization.^12^

DivJ kinase phosphorylates DivK, whereas PleC is the major phosphatase of DivK∼P (green box in Figure 2).^2^^,^^30^ In addition, PleC can function as a kinase to phosphorylate DivK. The kinase conformation of PleC is stimulated by interacting with DivK∼P.^23^ While phosphorylated DivK accumulates at the old pole during most of the cell cycle, it is also localized at the new pole in the predivisional stage and released from the new pole after cytokinesis^30^ (Figure 1, red). Interestingly, DivJ and PleC are localized at opposite poles (at the stalk and flagellum, respectively) during the cell cycle of *Caulobacter* (Figure 1, orange and purple).^31^^,^^10^ The catalytic activity and polar localization of DivJ and PleC regulate the spatial phosphorylation and dephosphorylation of DivK.^2^ Consequently, DivK is phosphorylated at the old pole and dephosphorylated at the new pole, and after cytokinesis, DivK is unphosphorylated in the newly formed swarmer cell.

Chen et al.^3^ and Tropini and Huang^2^ have constructed mathematical models to simulate the development of an asymmetrical distribution of CtrA∼P in predivisional cells. Their models account for the phenotypes of relevant mutant cells and suggest that *Caulobacter* establishes robust asymmetry before cytokinesis. Subramanian et al.^22^^,^^32^ proposed an “inhibitor-sequestration” model, suggesting that new-polar DivK∼P is sequestered by PleC kinase, so that CckA retains its kinase activity at the new pole in the predivisional stage. Li et al.^33^ built a stochastic version of Subramanian et al.’s “inhibitor-sequestration” model, in order to investigate molecular fluctuations of cell cycle regulators.

Although these published spatiotemporal models provide an initial understanding of the mechanisms of spatial regulation and pattern formation in *Caulobacter*, they assume an initial non-uniform localization of DivJ and PleC, so the source of asymmetry remains elusive. The initial asymmetry is created by scaffolding proteins found at the old and new poles at the time of cytokinesis.^34^ The key regulators of CtrA in *Caulobacter* respond to three scaffolding proteins—PodJ, PopZ, and SpmX.^10^^,^^34^ PodJ is localized at the flagellum pole, SpmX at the stalk pole, and PopZ at both poles during the *Caulobacter* cell cycle (Figure 1, blue, violet, and green).^10^^,^^34^ PodJ and SpmX directly bind to PleC and DivJ, respectively, causing PleC to accumulate at the new pole and DivJ at the old pole.^10^^,^^11^ In addition, the localization of DivK, DivL, CckA, and CpdR is dependent on PopZ.^10^^,^^12^^,^^35^ Mutant analysis indicates the new-polar DivL is determined by PodJ.^36^ As PleC and DivL, recruited by PodJ at the new pole, are binding partner of DivK, PodJ likely influences the localization of DivK as well.

Several hypotheses have been proposed for the initial polar localization of scaffolding proteins.^34^ The nucleoid-occlusion hypothesis suggests that the poles, because they are devoid of chromosomes, provide sufficient space for the assembly of scaffolding protein complexes.^37^ Other suggestions attribute polar accumulation to negative membrane curvature,^38^^,^^39^ or lipid^39^^,^^40^ or peptidoglycan^41^ composition at the poles.

Chen et al.^42^ and Subramanian et al.^32^^,^^43^ attributed the polar localization of PopZ to a Turing-pattern mechanism (see STAR Methods/localization of scaffolding proteins based on an activator-substrate depletion mechanism for turing patterns). To prevent PopZ polymerization at the center of the cell, the published PopZ model^43^ posited an unknown nucleating factor at the new pole, which biased the accumulation of PopZ polymer to the poles. The nucleating factor is likely to be PodJ,^11^ a scaffolding protein that localizes to the new pole before PopZ polymerizes there. With this possibility in mind, we propose an “activator-substrate depletion” (A-SD) reaction-diffusion mechanism (see STAR Methods and Xu et al.^44^) for the initial localization of three scaffolding proteins—PopZ, PodJ, and SpmX. In the course of developing the model presented here, we first considered an “intermediate” spatiotemporal model^44^ to verify the feasibility of Turing-pattern-driven polarity. In the intermediate model, PodJ nucleates the polymerization of PopZ at the new pole, and PopZ nucleates SpmX polymerization at the old pole. At the old pole, SpmX recruits DivJ kinase, which phosphorylates DivK. At the new pole, PodJ recruits PleC phosphatase, which dephosphorylates DivK∼P. In this way, based on the intermediate model, we included the bifunctional activity of PleC and the phosphotransfer signaling pathways between DivK∼P and CtrA, in order to explore the transient new-polar accumulation of DivK∼P and the molecular mechanisms underlying the asymmetrical distribution of CtrA∼P between swarmer and stalked compartments. In the new model, DivK∼P, in turn, creates an asymmetrical distribution of CckA, unphosphorylated CpdR (CpdRu), and CtrA∼P between swarmer and stalked compartments. The spatiotemporal dynamics of CtrA∼P then governs the progression of the *Caulobacter* cell through its cycle of DNA replication, differentiation, and cell division. We summarize the key assumptions made for our new spatiotemporal model in Methods S1. For a full description, see the “model details” section of STAR Methods. Our mathematical model correctly accounts for the two signaling hubs at opposite poles of a wild-type cell and for the spatiotemporal distributions of all the downstream components of the wild-type cell division cycle. In addition, the model accounts for aberrant distributions of signaling proteins observed in a variety of mutant cells and predicts not yet observed distributions of DivK∼P and CtrA∼P in some mutant strains (see Table S1).

## Results and discussion

### A-SD Turing patterns accurately capture the spatiotemporal dynamics of scaffolding proteins

**Figure 3:**
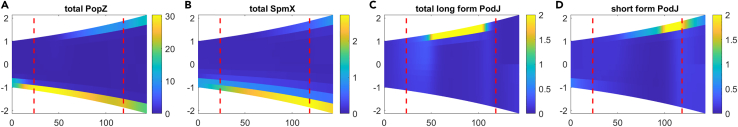
Spatial dynamics of scaffolding proteins over one cell cycle of a simulated wild-type *Caulobacter* cell (A–D) PopZ, SpmX, long-form PodJ, and short-form PodJ. Color denotes the scaled concentration of the indicated species; x axis is time (min), and y axis is distance from the midpoint of the long-axis of a cell. Each simulation starts (*t* = 0) with a newborn swarmer cell, with old pole at *y* = −1 and new pole at *y* = +1. The red dashed lines indicate the times of chromosome replication initiation (first) and cell compartmentalization (second).

The simulated spatial dynamics of the scaffolding proteins PopZ, SpmX, and PodJ in WT cells are shown as heatmaps in Figure 3. PopZ is localized at the old pole throughout the cell cycle. At approximately 50 min, a second focus of PopZ appears at the new pole (Figure 3A), matching experimental data.^11^ SpmX, recruited by PopZ, sharply accumulates at the old pole at 10–20 min in our simulation (Figure 3B), which agrees with experimental observations.^10^ Long-form PodJ (PodJL) polymerizes at the new pole during S phase (Figure 3C). There it is truncated by the protease PerP into the short form, PodJS (Figure 1, blue).^11^^,^^45^ PodJS remains at the flagellated pole until it is degraded during the sw-to-st transition of the next cell cycle (Figure 3D). Total PodJ (PodJL+PodJS) is evident at the new pole for most of the cell cycle (see Figure S1), as observed by Chen et al.^45^ and Zhao et al.^11^ The new cell synthesizes PodJL, which localizes to the new pole because old polar SpmX inhibits the polymerization of PodJL. These simulation results are consistent with experimental observations.

### Asymmetrical distributions of CtrA∼P and DivK∼P are reproduced by our model

The polar accumulation of scaffolding proteins induces spatial distributions of many other proteins in the signaling network. For example, the scaffolding proteins recruit DivJ and PleC to opposite poles of the cell, where they function, respectively, as a kinase and phosphatase of DivK, thereby creating an asymmetrical distribution of DivK∼P across the cell. Subsequently, DivK∼P binds to DivL to promote the switch of CckA from kinase to phosphatase.^46^^,^^47^ The CckA balance between kinase and phosphatase regulates the phosphorylation of CtrA and CpdR, thereby controlling the phosphorylation state and level of CtrA in the cell. In addition, PopZ binds to DivL and CpdR,^12^ and PodJ participates in the localization of DivL.^36^^,^^48^ Because the consequences of scaffolding-protein localizations are multifaceted and difficult to comprehend with confidence by informal reasoning alone, we include the detailed molecular mechanism of signaling modules in this work (green and yellow boxes in Figure 2) and explore the relationship between signaling proteins and scaffolding proteins.

**Figure 4:**
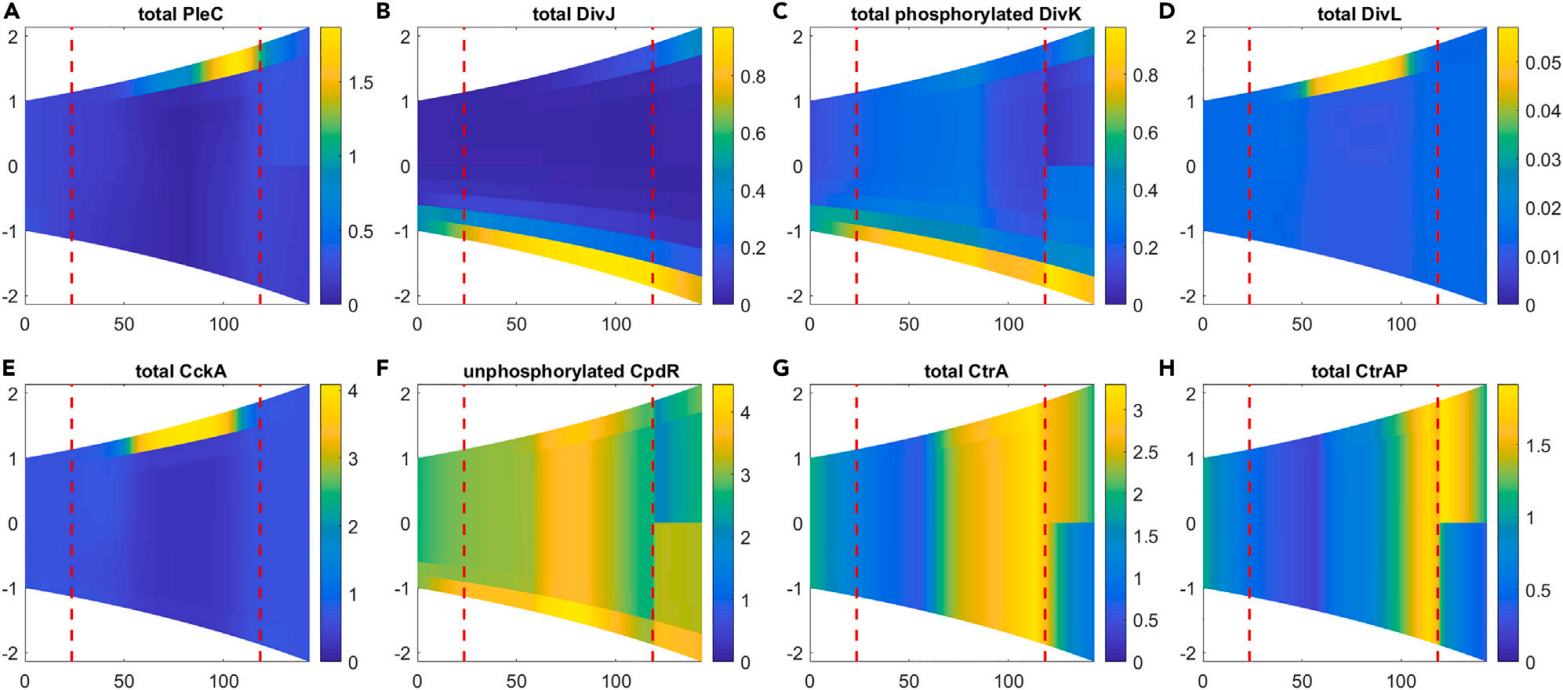
Spatial dynamics of client proteins in a simulated wild-type cell (A–H) PleC, DivJ, total DivK∼P, DivL, CckA, unphosphorylated CpdR, total CtrA, and CtrA∼P. Other details are identical to Figure 3.

Figure 4 shows the simulated dynamics of proteins involved in two phosphotransfer modules. Overall, the simulations match well with experimental observations. For example, DivK∼P accumulates in the stalked compartment after cell division. DivK∼P remains localized at the old pole (the stalk pole) over the entire cell cycle,^30^ while also showing temporary accumulation at the new pole in the predivisional stage.^30^ Our simulation captures the behavior of DivK∼P, although the temporary new-polar accumulation of DivK∼P is limited (Figure 4C). The asymmetric distribution of DivK is caused by new-polar PleC and old-polar DivJ, which regulate both the phosphorylation state and spatial localization of DivK (Figures 2 and 4A and B).

Experiments show that DivL is located at the new pole during S phase and less frequently at the old pole (Figure 1, olive).^49^ Our model captures the new-polar accumulation of DivL, which is mainly recruited by PodJ (Figure 4D). Similarly to DivL, CckA is dispersed in the nascent swarmer cell and shows strong and stable new-polar accumulation later,^14^^,^^24^ which is reproduced by our model (Figure 4E). A portion of *Caulobacter* cells (∼30%) exhibits old polar accumulation of CckA after the sw-to-st transition, which is not requisite for WT cell cycle. The total level of CpdR does not change much, while the phosphorylated CpdR level starts to increase in the predivisional stage (Figures S2G and S2H). Following cell division, unphosphorylated CpdR accumulates in the stalked compartment (Figure 4F). Our simulations of CpdR species are consistent with experiments.^13^ Phosphorylated CtrA accumulates in the swarmer compartment (Figure 4H), while unphosphorylated CtrA accordingly goes to the stalked compartment and subsequently is degraded. (Figure S2I). Because unphosphorylated CpdR promotes the proteolysis of CtrA, the asymmetric distribution of CpdR reinforces a reduced level of CtrA∼P in the stalked compartment, allowing the stalk cell to re-enter the DNA replication cycle.

A few studies suggest that CtrA∼P develops a spatial gradient in the predivisional stage by assuming CckA kinase and phosphatase are localized at the flagellated and stalked pole^3^^,^^22^; however, there has been no direct observation of a spatial gradient of CtrA∼P across a predivisional cell. Our model exhibits a different pattern, suggesting that a spatial gradient of CtrA∼P is not necessary for the development of asymmetry. In our model, the spatiotemporal dynamics of PleC and DivJ are sufficient to create an asymmetrical distribution of CtrA∼P between swarmer and stalked daughter cells. We show the dynamics of specific species defined in our model in Figure S2, including PleC kinase and phosphatase, DivL unbound to DivK∼P and DivL:DivK∼P complex, as well as CckA kinase and phosphatase. With the increase of DivJ level, DivK∼P increases and a portion of DivK∼P binds to PleC at the new pole at the predivisional stage, which turns a part of PleC into kinase (Figures 4B and 4C and S2A). With the chromosome replication fork passing *pleC*, [PleC] increases and PleC phosphatase dominates to dephosphorylate DivK∼P into unphosphorylated DivK at the new pole (Figure S2B). Therefore, [DivK∼P] at the new pole decreases before cell division ((Figure 4C). In addition, DivK∼P binds to DivL, which stimulates the phosphatase activity of CckA (Figure 2). Therefore, in our simulation, CckA phosphatase co-localizes with DivL:DivK∼P complex (Figures S2D and S2F). With the decrease of DivK∼P at the new pole, some DivL:DivK∼P dimers dissociate into DivL and DivK∼P, and CckA phosphatase is turned into kinase (Figures S2E) and S2F). After Z-ring closure, the localized CckA becomes dispersed throughout the cell due to the delocalization of DivL. At this time, most DivK∼P is found in the stalked daughter cell, where DivK is phosphorylated by old-polar DivJ. DivL:DivK∼P in the stalked daughter cell favors CckA phosphatase activity there. The flagellated daughter cell, meanwhile, maintains a high CckA kinase/phosphatase ratio. Therefore, CckA kinase and phosphatase exhibit an asymmetrical distribution between the swarmer and stalked compartments (see Figures S2E and S2F). CckA kinase and phosphatase are responsible for the phosphorylation and dephosphorylation, respectively, of both CpdR and CtrA. Given the asymmetrical distribution of CckA kinase and phosphatase, [CpdRu] and [CtrAu] are higher in the stalked daughter cell (see Figures 4F and S2I; the subscript “u” specifies the unphosphorylated form), and [CpdR∼P] and [CtrA∼P] are higher in the swarmer daughter cell (Figures 4H and S2H). In addition, both phosphorylated and unphosphorylated forms of CtrA are degraded by unphosphorylated CpdR, resulting in a low level of CtrA in the stalked daughter cell (Figure 4G). Taken together, the signaling pathway from PleC/DivJ to CckA explains why, in our model, CtrA (in its phosphorylated form) is distributed primarily to the new swarmer cell, even though there is no obvious gradient of CtrA∼P across the predivisional cell. Because CtrA∼P inhibits chromosome replication, the swarmer cell cannot replicate DNA, while the stalked cell (which has little CtrA) can initiate DNA replication immediately.

### Temporal dynamics of signaling proteins are also successfully reproduced by our model

**Figure 5:**
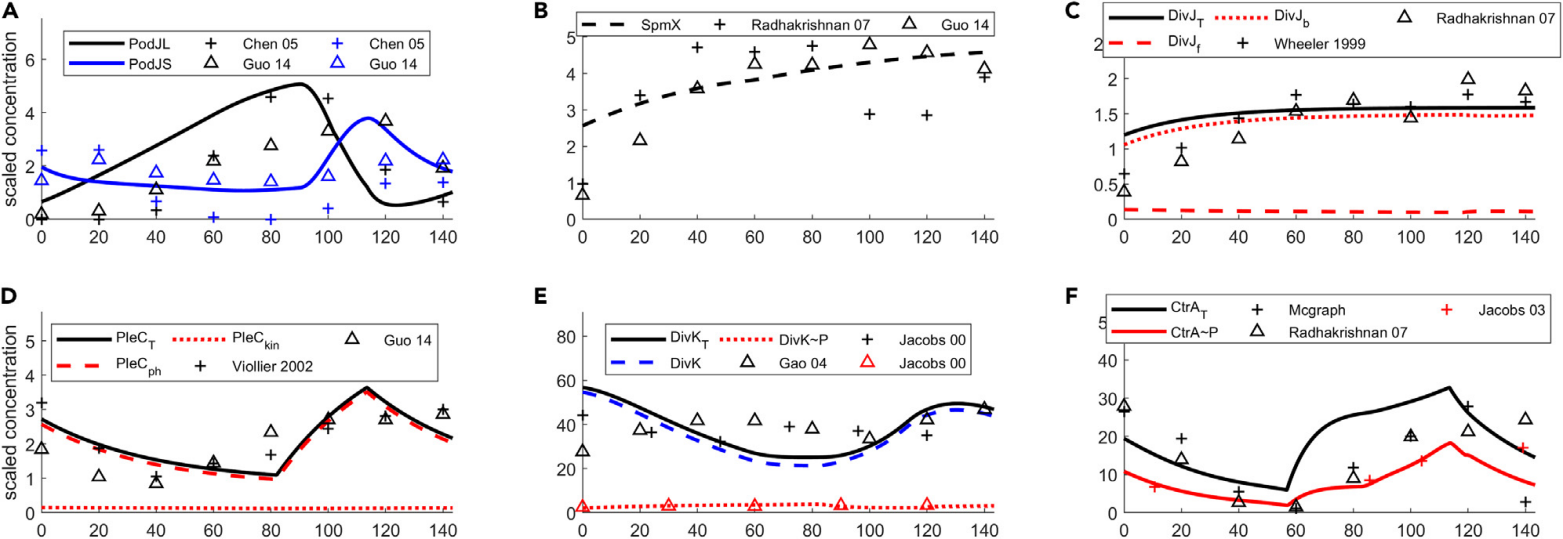
Comparison of simulated temporal dynamics with experimental observations over one wild-type cell-division cycle. X axis is time (min) from the birth of a swarmer cell; y axis is scaled concentration of proteins (A–F) PodJL and PodJS, SpmX, DivJ, PleC, DivK, and CtrA. Triangle and plus are experimental data points. Data collected as follows: [PodJL] and [PodJS] (Chen et al., 2005^50^; Guo, 2014^51^); [SpmX] (Radhakrishnan et al., 2008^10^; Guo, 2014^51^); [DivJ]_T_ (Wheeler and Shapiro, 1999^31^; Radhakrishnan et al., 2008^10^); [PleC]_T_ (Viollier et al., 2002^52^; Guo, 2014^51^); [DivK]_T_ (Guo, 2014^51^; Jacobs et al., 2001^53^); [CtrA]_T_ (McGrath et al., 2006^54^; Radhakrishnan et al., 2008^10^); CtrA∼P (Jacobs et al., 2003^55^).

In Figure 5, we compare the temporal dynamics of PodJL, PodJS, SpmX, DivJ, PleC, DivK, and CtrA with experimental measurements. In general, our temporal simulations fit the data quite well. PodJL increases steadily during the cell cycle, until PerP is expressed in late S phase, converting PodJL into PodJS (Figure 5A). Subsequently, PodJS is degraded at the beginning of the next cell cycle. SpmX level increases slowly throughout the cycle. Most of SpmX is in the stalked compartment at the end of cycle, which explains the low level of SpmX at the birth of a swarmer cell (Figure 5B). Similarly, because most DivJ is localized at the old pole, the nascent swarmer cell inherits less DivJ, which explains the lower level of DivJ at t = 0 (Figure 5C). In experiments, PleC drops during the swarmer stage, and then rises steadily in the stalk and predivisional cell. Simulated PleC level drops and rises as well, but the turning point occurs significantly later in the cell cycle (Figure 5D). The decrease of CtrA∼P (Figure 5F) at the sw-to-st transition signals the initiation of DNA replication and methylation-regulated gene expression in this model (see model details, Table S2). In our simulation, DNA replication commences approximately 25 min after cell separation.

### Interactions among scaffolding proteins and higher polar affinity are required for their proper spatial localization

**Figure 6:**
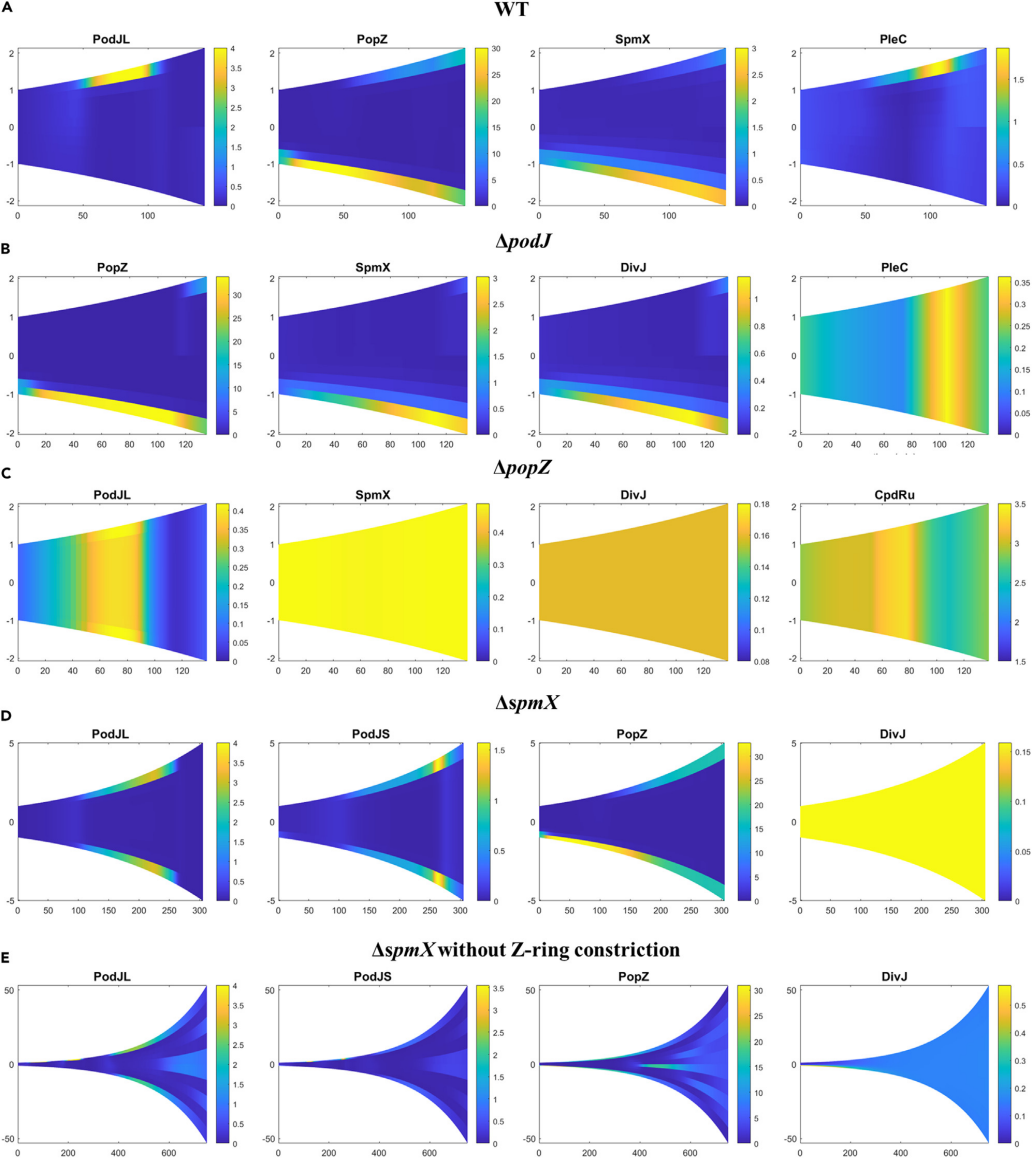
Spatial simulations of scaffolding proteins in WT, *ΔpodJ*, *ΔpopZ*, and *ΔspmX* mutant strains (A) WT background simulation. (B–D) Simulations of *ΔpodJ*, *ΔpopZ*, and *ΔspmX* strains with Z-ring constriction. (E) Simulation of *ΔspmX* without Z-ring constriction.

Because PopZ is a critical scaffolding protein recruiting core regulators of CtrA, such as SpmX, DivL, and CpdR,^12^ it is imperative to look into the mechanisms underlying PopZ localization.^11^ While PopZ shows bipolar accumulation in WT cells, no detectable PopZ accumulates at the new pole in *ΔpodJ* mutant cells, suggesting that PodJ is required to recruit PopZ to the new pole.^11^ We model *ΔpodJ* mutant cells by setting the synthesis rate of PodJ to 0 (Table S3). In the WT simulation, PodJ biases PopZ to bipolar accumulation (Figure 6A ). In a preliminary simulation of the *ΔpodJ* strain, polar localization of PopZ is drastically impaired (not shown), resulting in ectopic midcell accumulation of PopZ. These simulations suggest that, in addition to the clear role played by PodJ in stabilizing the polar localization of PopZ in WT cells, there is some other mechanism, independent of PodJ, that biases PopZ to polymerize at poles rather than midcell in *ΔpodJ* cells. For instance, the curvature of polar cell walls may provide higher affinity for scaffolding proteins like PopZ.^34^ Since the specific mechanism for polar accumulation of PopZ (independent of PodJ) is unclear, we simply assume a higher polymerization rate of PopZ at the poles. Specifically, the autocatalytic polymerization rate of PopZ (*k*_aut, PopZ_) is 25% larger in polar compartments than in central compartments, and similarly for PodJ. With this assumption of higher polar affinities, our model reproduces the phenotype of *ΔpodJ* cells, namely, that PopZ localizes solely to the old pole and PleC is dispersed uniformly across the cell throughout the division cycle (Figure 6B). In addition, SpmX and DivJ co-localize with PopZ in the *ΔpodJ* simulation.

In our simulation of *ΔpopZ* mutant cells (*k*_s,PopZ_ = 0), the polar localization of SpmX, DivJ, CpdR, and DivK is severely impaired (Figures 6C and S3), in agreement with experimental observations,^35^^,^^56^ although the remaining localized SpmX in Figure 4B of Bowman et al.^35^ is not reproduced. Furthermore, PodJ shows slight bipolar accumulation (Figure 6C), consistent with observations of Zhao et al.^11^ and Bowman et al.^35^ The delocalization of DivJ is likely caused by the delocalization of SpmX. Moreover, the bipolar localization of PodJ is likely a result of delocalized SpmX, because SpmX is an inhibitor on PodJ localization.^11^ In general, *ΔpopZ* cells, though viable, are severely impaired, exhibiting defects in cell division.^57^

To further investigate the function of SpmX, we set *k*_s,SpmX_ = 0 to simulate *ΔspmX* cells (Table S3). In this simulation, PodJ exhibits bipolar localization, while DivJ is dispersed (Figure 6D). Observations of *ΔspmX* cells reveal an increased number of cells with bipolar PodJ and ectopic midcell PodJ.^11^ Thus, our model captures certain properties of *ΔspmX* cells. As described in (STAR Methods/chromosome replication, methylation, and cell division), we do not model the process of Z-ring constriction; instead, we artificially introduce Z-ring constriction at 95 min after the initiation of chromosome replication (Figure S4). Assuming that the Z-ring does not close in *ΔspmX*, we provide, in Figure 6E, a 750 min simulation of *ΔspmX*, which shows that, without Z-ring closure, PodJ accumulates at poles and midcell in an elongated cell, as observed by Zhao et al.^11^ The agreement between Figure 6E and experiments suggests that Z-ring constriction is impaired in *ΔspmX* mutant cells.

Altogether, our A-SD Turing model reproduces most phenotypes of mutant strains deleted of scaffolding proteins, suggesting that our hypothesized interactions among PopZ, PodJ, and SpmX are crucial for their correct localization. Our analysis of these mutant strains suggests that PopZ and PodJ likely have a particular affinity for polar regions and higher rates of autocatalytic polymerization at the poles. Jacob et al. have proposed that the cell wall only grows in the central zone of bacterial cells,^58^ and this hypothesis has been supported by subsequent evidences.^59^^,^^60^^,^^61^ The spatially regulated synthesis and hydrolysis of peptidoglycan have been indicated to determine the curvature of *Caulobacter* cells.^62^^,^^63^ The unique curvature and less new wall insertion at cell poles may cause the higher affinity of PodJ and PopZ with poles suggested in this study. Using a two-dimensional model of a *Caulobacter* cell, Chen et al.^42^ have shown that the curvature of polar zones can bias PopZ polymerization there. Another possibility is that other proteins, such as ZitP and TipN, stabilize the polar accumulation of PopZ.^64^

### Our model captures key features of *Caulobacter* overexpression mutants

**Figure 7:**
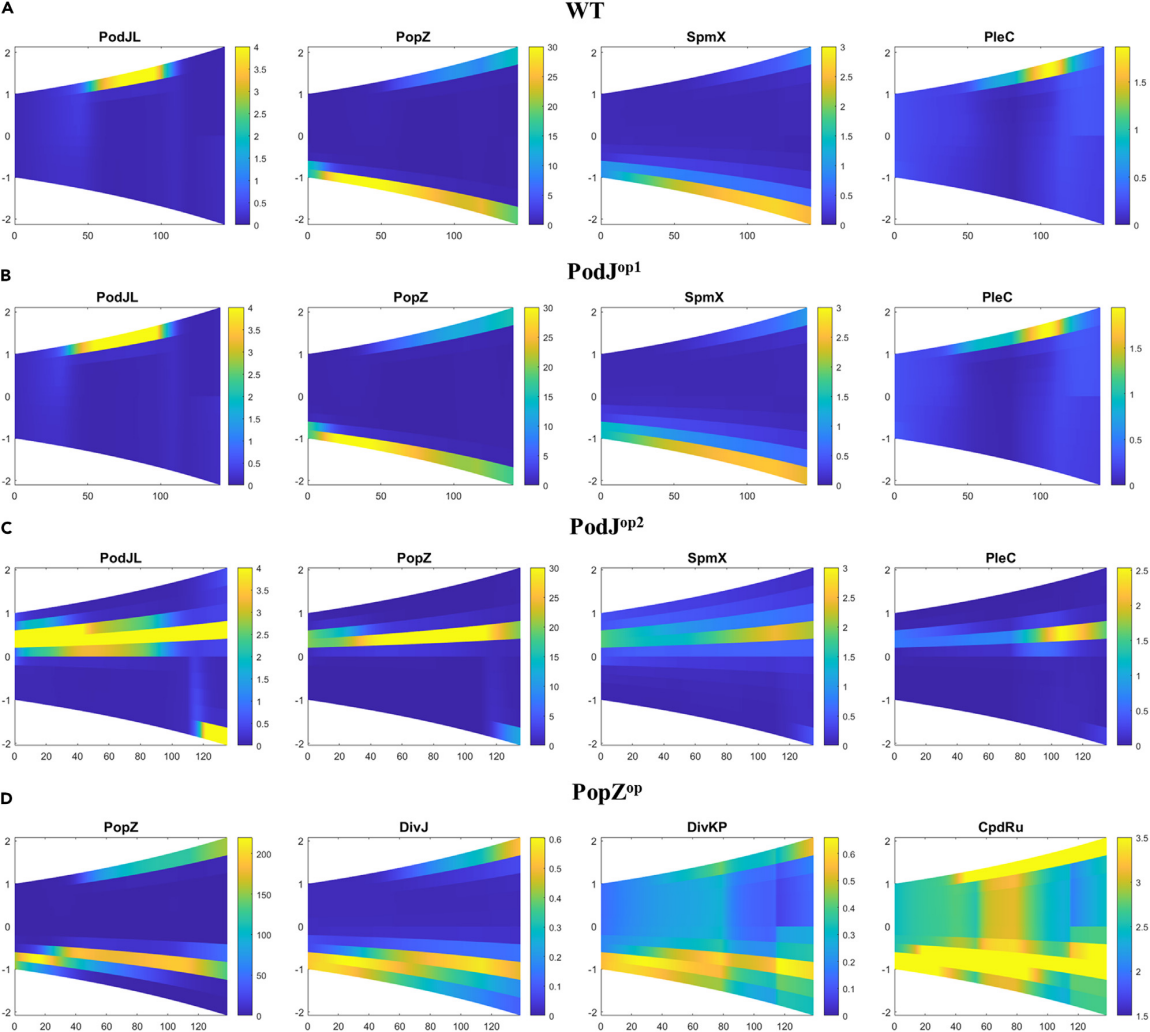
Spatial simulations for PopJ^op1^, PodpJ^op2^, and PopZ^op^ (A) WT cells; (B–D) overexpression mutant strains.

The phenotypes of mutant strains that overexpress scaffolding proteins provide additional tests of our model. For example, Zhao et al.^11^ provide experimental observations of *Caulobacter* cells overexpressing PodJ at two induction levels. Compared to WT cells, cells that mildly overexpress PodJ (0.03% xylose, Figure 4 in the study by Zhao et al.^11^) exhibit early accumulation of PopZ at the new pole. Greater overexpression of PodJ (0.3% xylose) creates elongated cells with multiple PodJ foci and branching morphologies with polar PodJ foci (Figure. S1 in the study by Zhao et al.^11^). To simulate these mutant cells, we overexpress PodJ at two different levels in our model; see the parameter settings (PopJ^op1^ and PopJ^op2^) given in Table S3. In the case of mild overexpression (PopJ^op1^, see Figure 7B), PodJL and PopZ exhibit early accumulation at the new pole, as observed. For greater overexpression (PopJ^op2^, see Figure 7C), PodJ is localized at poles and midcell, which is consistent with experiments. However, the cell division defect of cells that strongly overexpress PodJ is not captured by our model, because we do not consider the effects that overexpressed PodJ may have on the Z-ring constriction process.

In addition, we have considered the phenotype of mutant cells overexpressing PopZ (Table S3). These simulated cells (Figure 7D) display an expanded localization pattern for DivJ, DivK∼P, and CpdR, as observed in cells overexpressing PopZ at 0.3% xylose induction level (see Figures 4 and 5 in the study by Bowman et al.^35^).

### Our model captures the localization of DivK in existing mutant strains and predicts the phenotypes of novel mutations

Previous study has shown that phosphorylated DivK preferentially localizes at cell poles.^1^ Among the currently known binding partners of DivK, PleC and DivL show significantly higher affinity for DivK∼P *in vivo*, whereas DivJ binds to both phosphorylated and unphosphorylated forms.^46^ Therefore, we consider the following complexes of DivK in our model: PleC:DivK∼P, DivL:DivK∼P, DivJ:DivK∼P, and DivJ:DivK (see Figure 2). With these assumptions, our model reproduces the phenotypes of DivK localization in DivJ- and PleC-mutated cells, as described below.

**Figure 8:**
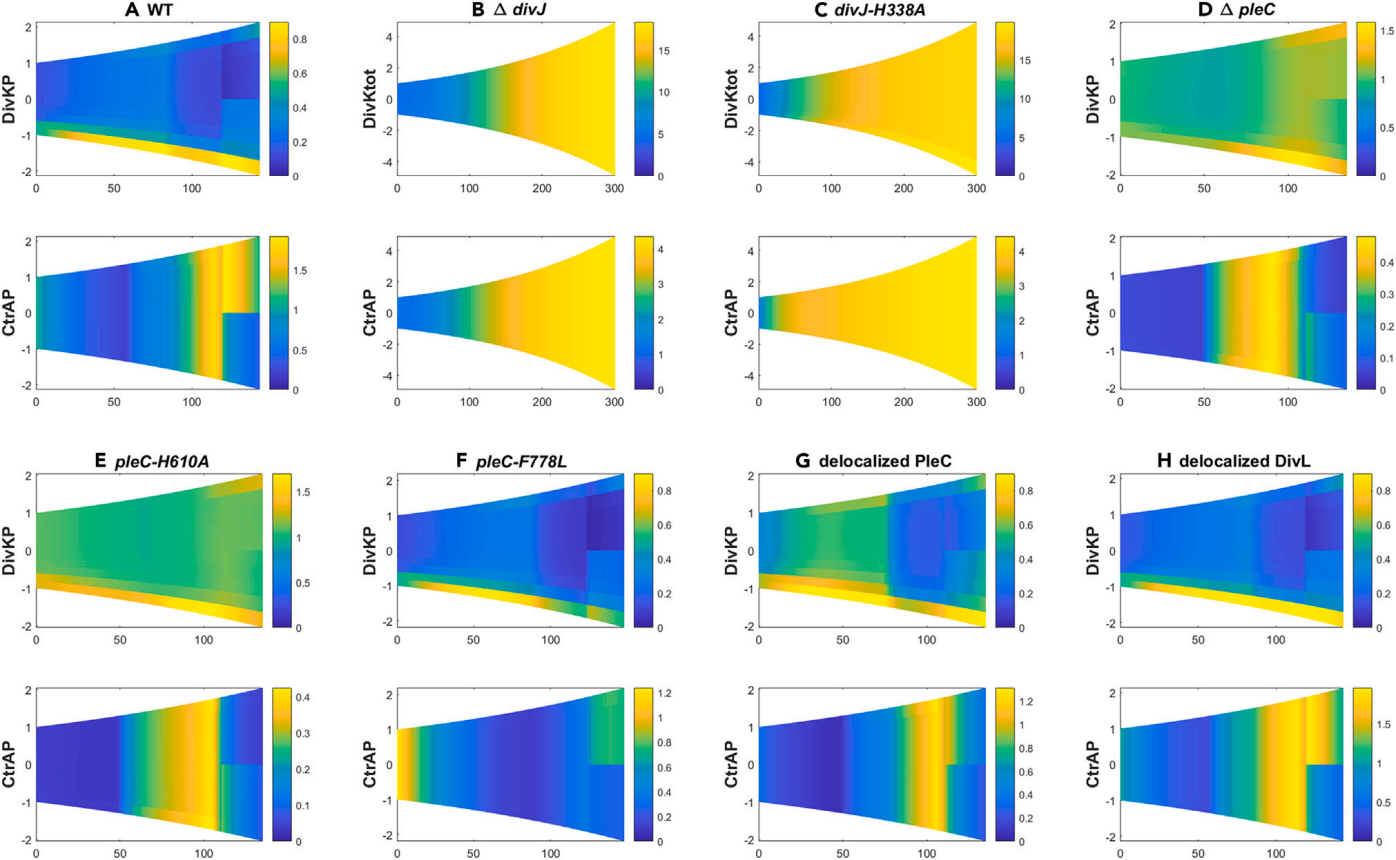
DivK(∼P) and CtrA∼P dynamics in WT and mutant strains (A–H) WT, *ΔdivJ*, *divJ-H338A*, *ΔpleC*, *pleC-H610A*, *pleC-F778L*, delocalized PleC, and delocalized DivL. Total DivK, rather than DivK∼P, is plotted for the two divJ-mutant strains because DivJ can bind to unphosphorylated DivK while PleC and DivL prefer to bind to phosphorylated DivK. In “delocalized PleC” and “delocalized DivL” simulations, the recruitment by PodJ on PleC and DivL is deleted, respectively.

DivJ is necessary for the polar localization of DivK. DivJ not only binds to DivK but also is the major kinase phosphorylating DivK.^1^ Without DivJ (see the *ΔdivJ* mutant, Table S3), the level of DivK∼P drops dramatically and DivK is delocalized.^1^^,^^7^^,^^53^ In the kinase-defective DivJ strain (*divJ-H338A*), DivK can localize at the old pole but fails to localize at the new pole.^1^ Our simulations of *ΔdivJ* and *divJ-H338A* (Figures 8B and 8C) are consistent with these observations, which suggest that DivJ determines the old-polar localization of DivK and that DivJ kinase activity is required for the new-polar accumulation of DivK. On the other hand, the viability of *ΔdivJ* cells calls into question our simulation of uniformly high levels of CtrA∼P across a dividing cell, which would block DNA replication in the progeny.

PleC is believed to determine the release of DivK from the new pole rather than its localization there, because DivK∼P continues to occupy the new pole after Z-ring closure in the PleC-deficient (*ΔpleC*) and the catalytically inactive (*pleC-H610A*) mutant strains.^1^^,^^21^^,^^30^ Our simulations of *ΔpleC* and *pleC-H610A* in Figures 8D and 8E show that DivK∼P fails to release from the new pole after cell division. We have also simulated the kinase-defective *pleC* strain *pleC-F778L*, in which DivK shows WT-like dynamics (Figure 8F), consistent with experiments.^30^ These results suggest that the kinase activity of PleC is dispensable for the transient new-polar accumulation of DivK in the predivisional stage.

Based on these results, we speculate that either PleC or DivL (or both) functions as the physical binding partner to recruit DivK∼P to the new pole, which also requires the kinase activity of DivJ. To further explore the roles of PleC and DivL, we use our model to predict the phenotypes of two hypothetical mutant cases, “delocalized PleC” (*k*_fb,PleC_ = 0, knocking out the recruitment of PleC by PodJ, which might correspond to mutant cells expressing PodJ_PSE, because the PSE domain is required for PleC recruitment to the cell pole in *E. coli*^11^) and “delocalized DivL” (*α*_DivLPodJ_ = 0, knocking out the recruitment of DivL by PodJ; which might correspond to mutant cells deleted for MopJ, the mediator between PodJ and DivL;^36^ alternatively, expressing a mutant PAS domain in PodJ, because the PAS domain commonly serves as binding site for protein-protein interactions^48^). When PleC is delocalized, more DivK∼P is likely bound to DivL at the new pole. In the simulation of delocalized DivL mutant, DivK∼P shows WT-similar distribution. Therefore, we suggest that both DivL and PleC are able to recruit DivK∼P at the new pole.

To sum up, our model agrees with experimental observations that DivJ kinase activity is required for new-polar accumulation of DivK, while PleC is required for the timely release of DivK from the new pole.^1^ In addition, our mutant simulations suggest that PleC localization and kinase activity are not necessary for the localization of DivK.

### Our model captures key characteristics of phosphotransfer processes

In *ΔdivJ* cells, the level of DivK∼P is reduced and CtrA-dependent transcriptions increase,^7^^,^^65^ and these properties are successfully recapitulated by our model (Figure 8B). Our simulation of the *ΔpleC* strain shows increased level of DivK∼P and reduced level of CtrA∼P (Figure 8D), which is consistent with experiments as well.^25^^,^^65^ In Table 1, we compare simulated levels of DivK∼P to corresponding experimental measurements in four mutant strains. Our simulations capture the key trends of DivK∼P level in these mutant cases. DivK∼P level dramatically decreases in *ΔspmX* and *ΔdivJ* because the kinase activity of DivJ is largely impaired or deleted (Table 1, Figures S3 and 8B). As PleC mainly functions to dephosphorylate DivK, *ΔpleC* mutation results in increased DivK∼P (Table 1 and Figure 8D). In addition, the higher level of DivK∼P in *ΔpodJ* suggests that PodJ likely inhibits rather than activates the kinase activity of PleC, which is a debatable issue (see the study by Kowallis^34^).

**Table 1:** Phosphorylation state of DivK in mutant strains

mutant		WT	*ΔspmX*	*ΔdivJ*	*ΔpleC*	*ΔpodJ*
DivK∼P^a^	Exp^b^	1	0.36	0.04	1.79	1.50
	Sim	1	0.28	0.09	3.34	1.64

a DivK∼P levels are normalized with respect to the level in wild-type cells.

b Experimental data of *ΔspmX*, *ΔdivJ*, and *ΔpleC* are collected from Radhakrishnan et al.^10^ and quantified using ImageJ. Experimental data of Δ*podJ* are collected from Guo.^51^

By interacting with DivL, DivK∼P inhibits phosphotransfer to CtrA∼P; hence, mutations that impact the localization and/or abundance of DivK∼P should affect the spatiotemporal dynamics of CtrA∼P. In this regard, our simulations of CtrA spatial dynamics for some relevant mutant strains (Figures 8 and S3) are subject to experimental verification. *ΔpleC* and *pleC-H610A* mutant strains, which fail to release DivK∼P from the new pole, exhibit reverse distributions of CtrA∼P in simulations (Figures 8D and 8E). Our model predicts the asymmetrical distribution of CtrA∼P almost disappears in *ΔdivJ*, *divJ-H338A*, *ΔpopZ*, and *ΔspmX* mutant strains. All other mutant simulations in this study show higher levels of CtrA∼P in the swarmer compartment, whereas the “delocalized PleC” and *ΔpodJ* mutant simulations display smaller differences of CtrA∼P distribution between swarmer and stalked compartment (Figures 8 and S3). These predictions suggest that both the activity and the distribution of regulators contribute to the establishment of asymmetry.

### Conclusion

To study the establishment of cell polarity and asymmetric division in the alpha-proteobacterium *C. crescentus*, we integrate a Turing-type model of spontaneous pattern formation in reaction-diffusion equations with a protein signaling network linking scaffolding proteins to phosphotransfer pathways. Symmetry breaking derives from an A-SD mechanism, where “substrate” is monomeric protein subunits and “activator” is polymeric protein, whose branched structure supports autocatalytic growth of the polymer. Quite naturally, the substrate molecules diffuse much more rapidly than the polymeric material, which is the primary requirement for developing distinct foci of polymerization (i.e., Turing patterns). In our model, the three scaffolding proteins (PodJ, PopZ, and SpmX) are polymerized based on the A-SD mechanism and interact with each other. Constructed in this way, our model accounts for the observed patterns of polymerization of scaffolding proteins at the two poles of a growing *Caulobacter* cell. Subsequently, the spatial distribution of the scaffolding proteins spatially influences the two phosphotransfer modules that ultimately control the accumulation and phosphorylation of the master transcription factor CtrA in the growing cell. Phosphorylated CtrA (CtrA∼P) is abundant in a newborn, motile swarmer cell. At the transition from the swarmer (flagellated) morphology to the sessile (stalked) morphology, CtrA activity is cleared by dephosphorylation and degradation. The flagellum is dropped and a stalk develops in its place at the “old” pole. The stalk cell initiates DNA replication and eventually generates a flagellum at the opposite end (the “new” pole) of the cell. In the predivisional cell, CtrA is resynthesized and phosphorylated by CckA-kinase activity at the new pole. After the Z-ring closes, CtrA∼P is found only in the nascent swarmer cell (with kinase activity). In the nascent stalk cell, CtrA is degraded by a CpdR-dependent protease. Eventually, the cell divides asymmetrically into a sessile stalk cell (mostly devoid of CtrA) and a motile swarmer cell (with abundant CtrA∼P).

The network of biochemical reactions that controls these processes of spatial differentiation and temporal development, as it has been worked out by molecular cell biologists over past decades, is exceedingly complex. Even a simplified, partial description of the network consists of dozens of proteins in different phosphorylation states and in complex with different partners (see Figure 2). By informal reasoning alone, it is impossible to know how successful this conception of the control system might be in explaining known properties of *Caulobacter* cell replication, in accounting for phenotypes of many regulatory mutations, and in predicting the behavior of cells under novel circumstances. To answer these questions requires the precision of a mathematical model based on well-known properties of the control system, calibrated against quantitative experimental observations, tested by its predictions of known mutant phenotypes, and (ultimately) pushed to make novel predictions that can be tested experimentally.

A mathematical model of such detail is necessarily complex, consisting of 39 differential equations (see Methods S2) involving 110 parameters (Table S4), of which 41 had to be estimated from experimental observations of protein time courses and spatial distributions. These parameters were estimated from data on a few key species in wild-type cells. Then the model (with the estimated parameter values) was verified by comparing simulations with the whole set of available data on protein abundances and distributions in wild-type cells and a variety of well-studied mutant strains. The surprisingly good match between model simulations and experimental data demonstrates the strength of the model.

### Limitations of the study and future directions

Although our model reproduces many key characteristics of experimental observations, there are some notable discrepancies between the model and some mutant phenotypes. For example, our model cannot explain the initiation of DNA replication in *ΔdivJ* cells^31^ or the deviant division patterns of *ΔpopZ* cells.^57^ Reconciling some of these discrepancies may lead to a more accurate and predictive mathematical model in the future. Some differences may stem from the fact that our model does not explicitly track the regulation of DNA synthesis, of chromosome methylation, of Z-ring closure, or of cell separation. Other limitations derive from the current parameterization procedure, which is not highly efficient for simulating mutant strains and cannot evaluate the robustness of the model. To improve and extend our model, we see four future directions.

1. Integrate this spatial model with previous temporal models of the CtrA-DnaA-GcrA-CcrM regulatory network to investigate the comprehensive control of DNA replication and chromosome methylation.
2. Supplement the model with interactions among PopZ, ParA, ParB, and FtsZ to account for the spatial regulations of chromosome segregation and cell division.
3. Extend the parameter estimation-verification procedure to derive a representative distribution of parameter sets that all provide “reasonably” good fits to the constraining datasets, in order to assess the robustness of the model and to estimate the reliability of predictions made by the model across the range of parameters sets.
4. Convert the deterministic model into a stochastic version to capture the inherent variability of bacterial cell development.

## STAR★Methods

### Key resources table

**Table:** 

REAGENT or RESOURCE	SOURCE	IDENTIFIER
**Software and algorithms**		

ImageJ	https://doi.org/10.1038/nmeth.2089	https://imagej.net
MATLAB	The MathWorks Inc.	R2020a

**Other**		

Caulobrowser	Lasker et al.,2016^66^	http://web.stanford.edu/group/golden gate don/cgi-bin/index/index.py
Codes related to the spatiotemporal model	https://github.com/chunruixu/Turing-pattern-model-of-scaffolding-proteins.git	

### Resource availability

#### Lead contact

Further information and requests for resources and reagents should be directed to and will be fulfilled by the lead contact, Yang Cao (ycao@cs.vt.edu).

#### Materials availability

This study did not generate new unique reagents.

**Experimental model and subject details**

No new experiments are reported in this study.

### Method details

#### General simulation methodology

All simulations and quantitative analyses were conducted with customized MATLAB R2020a scripts. PDEs are converted into ODEs through a compartment-based discretization (see the next section ‘reaction-diffusion equations and compartment-based simulation’). ODEs are solved by MATLAB’s ode15s. A simulated cell cycle starts from a new-born swarmer or stalked cell (*t* = 0) and ends when the incipient daughter cells are separated completely. The nascent daughter cell is reassigned to *N* compartments (*N* = 10 in this study, unless otherwise specified) for simulating the next cell cycle. Because most experimental data are measured on a synchronized swarmer cell population,^67^ simulations in this study are for swarmer cells, for the most part. Some representative simulations of stalked cells are provided in Figure S5.

We first select a set of pseudo initial values of variables based on experimental observations. For example, PopZ polymers accumulate at the old pole at the beginning of the cell cycle, so the initial concentration of [PopZ_p_] in the old-polar compartment has to be higher than other compartments. We run simulations with these pseudo initial values, estimate parameters with the genetic algorithm, and replace the initial condition by initial values at the beginning of the second simulated cell cycle. Details of the pseudo initial values can be obtained from our codes at https://github.com/chunruixu/Turing-pattern-model-of-scaffolding-proteins.git.

All the results presented in the main text were calculated from a ten-compartment version of the PDEs in Methods S2 using the estimated parameter values in Table S4 for WT cells. Simulation time increases quadratically with compartment number (Figure S6). Ten-compartment is a suitable compromise between accuracy and efficiency of simulating the model.

To simulate mutant cells, we make appropriate changes to some of the parameters, as specified in Table S3. To wipe out any biases introduced by the initial conditions, the simulation is run for a few cell cycles to reach a stable, repetitive sequence of cell cycle events. For a WT cell or a mutant cell that initiates DNA replication within 300 min in our simulation, we plot results of the fifth cycle. For mutant cells that do not initiate DNA replication by 300 min, we plot simulated results of 0–300 min.

We qualitatively summarize the regulatory network of our model in Figure 2, the key assumptions in Methods S1, and the roles of individual regulators in Table S5.

#### Reaction-diffusion equations and compartment-based simulation

To model the spatiotemporal dynamics of a generic protein, we propose the reaction-diffusion equation

$$\frac{\partial C}{\partial t}=\text{Chemical}\;\text{Reaction}\;\text{Rates}+D\frac{\partial^{2}C}{\partial x^{2}}\text{,}$$

where *C(x*,*t)* is the concentration of the protein and *D* is its diffusion coefficient. Chemical Reaction Rates = rate of synthesis – rate of degradation – rate of dilution ± molecular interaction rates. By dividing the spatial domain into *N* compartments of length *l* = *L/N*, we convert this partial differential equation (PDE) into a set of *N* ordinary differential equations (ODEs) for *C*_*i*_(*t*), *i* = 1, ..., *N*. For a ten-compartment model,

$$\left\{ \begin{array}{c} \frac{dC_{1}}{dt}=\text{CRR}+\frac{D\left(C_{2}-C_{1}\right)}{l^{2}}\\ \frac{dC_{i}}{dt}=\text{CRR}+\frac{D\left(C_{i+1}-C_{i}\right)}{l^{2}}+\frac{D\left(C_{i-1}-C_{i}\right)}{l^{2}},i=2∼9\\ \frac{dC_{10}}{dt}=\text{CRR}+\frac{D\left(C_{2}-C_{1}\right)}{l^{2}} \end{array}\right.$$

The first compartment represents the new pole of a *Caulobacter* cell and the tenth compartment is the old pole. The number of compartments *N* influences the computational complexity of the model. For initially exploring the model and searching its parameter space, we used a simpler four-compartment model, described in Methods S3. After the model was initially verified and the parameters estimated, we extended the four-compartment scheme to ten compartments to explore the spatiotemporal dynamics of proteins and provide more accurate simulations, as presented in the main text.

To take into account the fact that a *Caulobacter* cell is growing as a result of new cell wall materials being added uniformly along the long axis,^68^ we assume each compartment grows exponentially with time (see below).

$$\frac{dl}{dt}=\mu l\text{.}$$

During the swarmer cell cycle, a swarmer cell grows, over the course of 150 min, from ∼2 μm at birth to ∼4.4 μm at separation,^69^^,^^70^ so we calculate $\mu =\frac{1}{150\;\min}\log\left(\frac{4.4}{2}\right)\approx 0.0053\;\min^{-1}\text{.}$

#### Localization of scaffolding proteins based on an activator-substrate depletion mechanism for turing patterns

In 1952, Alan Turing proposed a chemical reaction-diffusion model for the spontaneous generation of spatial patterns in an otherwise homogeneous reaction vessel.^71^ Since then, many authors have investigated the criteria for pattern formation in activator-inhibitor (AI) and activator-substrate depletion (A-SD) mechanisms.^72^^,^^73^^,^^74^ In short, the ‘activator’ must be produced autocatalytically, and the ‘inhibitor’ (or the ‘substrate’) must diffuse much faster than the activator. Furthermore, for the A-SD mechanism, the rate of the conversion of substrate into activator must be proportional to [substrate][activator]^*n*^, where *n* = 2 or larger.

In *Caulobacter* cells, the three scaffolding proteins (PodJ, PopZ and SpmX) satisfy these criteria for A-SD Turing pattern formation. These proteins have monomeric and polymeric forms, and the monomer subunit (the ‘substrate’) diffuses much faster than the polymer (the ‘activator’).^11^^,^^56^ Furthermore, PodJ, PopZ, and SpmX self-assemble *in vitro* and *in vivo*,^34^ and they grow by branching mechanisms.^11^^,^^43^^,^^56^^,^^75^^,^^76^

The branching mode of polymerization causes polymeric material to increase autocatalytically: as the polymer grows, new ends are created by branching, and consequently, new polymerization can proceed at an ever-increasing rate. If the total concentration of free ends increases linearly with the total concentration of polymer, [PopZ_p_], then we might expect that the rate of autocatalytic polymerization would be proportional to [PopZ_m_][PopZ_p_], but Turing pattern formation requires that the rate be proportional to [PopZ_m_][PopZ_p_]^2^.^77^ To justify the quadratic dependence on [PopZ_p_], we suggest that free ends might serve as catalysts for adding a monomer to a branching site on the side of a polymer chain, so that the rate of branching is proportional to [PopZ_m_][PopZ_fe_][PopZ_bs_] (‘fe’ is free end and ‘bs’ is binding site). Assuming, reasonably enough, that the concentrations of ‘free ends’ and ‘binding sites’ are each proportional to [PopZ_p_], we get the required dependence on [PopZ_p_]^2^.

An A-SD mechanism can exhibit unipolar, central, and bipolar accumulations of activator, depending on the wavelength of the Turing instability and the length of the cell.^43^ These characteristics of Turing patterns fit well with observations that PopZ has an old-pole focus in nascent swarmer cells and accumulates at both poles in longer stalked cells and predivisional cells (Figure 1). Furthermore, in mutant cells that fail to separate, the resulting filaments have spatially periodic zones of scaffolding protein polymerization, as expected of a Turing pattern.^11^^,^^56^^,^^76^ Hence, following Subramanian et al. (2014),^43^ we employ an A-SD Turing pattern model for the localization of PopZ and the other two scaffolding proteins, PodJ and SpmX. PopZ directly interacts with PodJ and the robust accumulation of PopZ at the new pole requires PodJ.^11^ Furthermore, PopZ is required for the proper localization of SpmX,^35^ and SpmX is an inhibitor of PodJ localization.^11^ So, we must take into account the interactions of three scaffolding proteins, all of which are likely polymerizing at the poles in response to A-SD mechanisms of Turing pattern formation.

With these factors in mind, we write reaction-diffusion equations for PopZ_m_ and PopZ_p_ as follows:

$$\frac{d\left[{\text{PopZ}}_{m}\right]}{dt}=k_{s,\text{PopZ}}-\left(k_{d,\text{PopZ}}+\mu \right)\cdot \left[{\text{PopZ}}_{m}\right]+k_{\text{depol},\text{PopZ}}\cdot \left[{\text{PopZ}}_{p}\right]-k_{\text{dnv},\text{PopZ}}\cdot \left(1+\alpha_{\text{PopZPodJ}}\cdot \left[{\text{PodJL}}_{T}\right]\right)\cdot \left[{\text{PopZ}}_{m}\right]-k_{\text{aut},\text{PopZ}}\cdot \left[{\text{PopZ}}_{m}\right]\;{\cdot \left[{\text{PopZ}}_{p}\right]}^{2}+D_{\text{PopZm}}\cdot \frac{\partial^{2}\cdot \left[{\text{PopZ}}_{m}\right]}{\partial x^{2}}$$

$$\frac{d\left[{\text{PopZ}}_{p}\right]}{dt}=-\left(k_{d,\text{PopZ}}+\mu \right)\cdot \left[{\text{PopZ}}_{p}\right]-k_{\text{depol},\text{PopZ}}\cdot \left[{\text{PopZ}}_{p}\right]+k_{\text{dnv},\text{PopZ}}\cdot \left(1+\alpha_{\text{PopZPodJ}}\cdot \left[{\text{PodJL}}_{T}\right]\right)\cdot \left[{\text{PopZ}}_{m}\right]+k_{\text{aut},\text{PopZ}}\cdot \left[{\text{PopZ}}_{m}\right]\;{\cdot \left[{\text{PopZ}}_{p}\right]}^{2}+D_{\text{PopZp}}\cdot \frac{\partial^{2}\cdot \left[{\text{PopZ}}_{p}\right]}{\partial x^{2}}$$

[PopZ_m_] and [PopZ_p_] are the concentration of PopZ monomer and polymer, respectively. [PodJL_T_] = [PopZ_m_] + [PopZ_p_], denoting the total concentration of long form PodJ. *k*_s,PopZ_ and *k*_d,PopZ_ are the rate constants for synthesis and degradation of PopZ. *k*_depol,PopZ_ is the rate constant for depolymerization, while *k*_dnv,PopZ_ and *k*_aut,PopZ_ are the rate constants for *de novo* and autocatalytic polymerization of PopZ, respectively. The parameter *α*_PopZPodJ_ describes the role of PodJ in recruiting PopZ for *de novo* polymerization. *D*_PopZm_ and *D*_PopZp_ are the diffusion coefficients of monomer and polymer of PopZ respectively, and *D*_PopZm_ ≫ *D*_PopZp_. The diffusion coefficients of all monomeric proteins in this study are estimated by an empirical function (shown below), derived for proteins diffusing in *E*. *coli*.^78^

$$D=\alpha {\left(MW\right)}^{-2}+D_{0}\text{,}$$

where $\alpha =4.3\times 10^{3}{\mu m}^{2}s^{-1}k{\text{Da}}^{2}$, D_0_ = 0.65μm^2^s^-1^ and *MW* is the molecular weight of the protein in kDa.

To derive PDEs for the accumulation of PodJ in the cell, we must take into account the inhibitory effect of SpmX on the localization of PodJ,^11^^,^^44^ see Figure 2 blue box. PodJ shows bipolar localization when it is expressed alone in *E*. *coli*, suggesting that PodJ has an intrinsically high affinity for both poles.^11^ Co-expression of SpmX and PodJ in *E*. *coli* results in dispersed PodJ.^11^ PodJ accumulates at both poles in *ΔspmX* mutant cells, and polar accumulation of PodJ is reduced in SpmX-overexpressed strains.^11^ Taken together, these observations suggest that SpmX suppresses the polymerization of PodJ (Figure 2 blue box). We include the inhibition effect of SpmX on PodJ polymerization in equations of PodJL, as follows:

$$\frac{d\left[{\text{PodJL}}_{m}\right]}{dt}=k_{s,\text{PodJ}}\cdot \left(\left(1-\epsilon \right)\cdot S_{podJ}+\epsilon \right)+k_{s,\text{PodJ}2}\cdot \frac{J_{i,\text{PodJCtrA}}^{4}}{J_{i,\text{PodJCtrA}}^{4}+{\left[\text{CtrA}∼P\right]}^{4}}-\left(k_{d,\text{PodJ}1}+k_{d,\text{PodJ}2}\cdot \left[\text{PerP}\right]+\mu \right)\cdot \left[{\text{PodJL}}_{m}\right]+k_{\text{depol},\text{PodJ}}\cdot \left[{\text{PodJL}}_{p}\right]-k_{\text{dnv},\text{PodJ}}\cdot \left[{\text{PodJL}}_{m}\right]-\frac{k_{\text{aut},\text{PodJ}}}{1+\alpha_{\text{PodJSpmX}}\cdot \left[{\text{SpmX}}_{T}\right]}\cdot \left[{\text{PodJL}}_{m}\right]\;{\cdot \left[{\text{PodJL}}_{p}\right]}^{2}+D_{\text{PodJLm}}\cdot \frac{\partial^{2}\cdot \left[{\text{PodJL}}_{m}\right]}{\partial x^{2}}$$

$$\frac{d\left[{\text{PodJL}}_{p}\right]}{dt}=-\left(k_{d,\text{PodJ}1}+k_{d,\text{PodJ}2}\cdot \left[\text{PerP}\right]+\mu \right)\cdot \left[{\text{PodJL}}_{p}\right]-k_{\text{depol},\text{PodJ}}\cdot \left[{\text{PodJL}}_{p}\right]+k_{\text{dnv},\text{PodJ}}\cdot \left[{\text{PodJL}}_{m}\right]+\frac{k_{\text{aut},\text{PodJ}}}{1+\alpha_{\text{PodJSpmX}}\cdot \left[{\text{SpmX}}_{T}\right]}\cdot \left[{\text{PodJL}}_{m}\right]\;{\cdot \left[{\text{PodJL}}_{p}\right]}^{2}+D_{\text{PodJLp}}\cdot \frac{\partial^{2}\cdot \left[{\text{PodJL}}_{p}\right]}{\partial x^{2}}$$

[SpmX_T_] = [SpmX_m_] + [SpmX_p_]. The parameter *α*_PodJSpmX_ controls the strength of SpmX inhibition of PodJ polymerization. *J*_i,PodJCtrA_ is the dissociation constant for CtrA∼P binding to the promoter of the *podJ* gene. CtrA∼P binding suppresses *podJ* expression. The factor ((1−*ϵ*)·*S*_*podJ*_ + *ϵ*) represents the regulation of *podJ* expression by DNA methylation, which is explained in the next section.

#### Chromosome replication, methylation, and cell division

In addition to CtrA, there are other proteins controlling the initiation of chromosome replication. One key regulator is DnaA, which binds to *Cori* to initiate replication.^15^ During replication, a fully-methylated chromosome becomes a pair of hemi-methylated chromosomes. CcrM, a DNA methyltransferase that is activated as replication is completed, remethylates promoters at specific methylation sites.^79^ In this study, we do not explicitly model the control of replication by GcrA and DnaA as well as CtrA and the methylation of chromosomes by CcrM because these events are not closely coupled to spatial regulations, although they are vital to temporal checkpoints during the cell cycle. We assume that DNA replication is initiated (time = *T*_ini_) when CtrA∼P drops below a threshold, Θ^20^ (Table S2). The replication period (S phase) of WT cells is approximately 90 min,^80^ so we set the termination time *T*_term_ = *T*_ini_ + 90 min. For promoters with methylation sites (*ctrA*, *pleC*, *perP*, and *podJ*), we use the factor ((1−*ϵ*)·*S*_*podJ*_ + *ϵ*) to model the effect of methylation (see yellow rectangles with ‘Meth’ in Figure 2):

• *S* = 0: fully-methylated promoters with lower rate of transcription.
• *S* = 1: hemi-methylated promoters.

where *ϵ* is a small number indicating the suppressed expression of genes when fully methylated (see Methods S2). Because bacterial genes are replicated in the linear order in which they are located on the chromosome,^81^ we set *S* = 1 at the time when the replication fork passes a gene, based on its genome coordinates.^82^^,^^66^ When replication terminates, *S* is set back to 0. The switching parameters for these events are listed in Table S2.

Z-ring closure is not modelled in this study. Instead, we assume that compartmentalization is completed (Z-ring closes) 5 min after DNA replication terminates. The swarmer and stalked cells separate (i.e., the simulated cell cycle completes) about 25 min after Z-ring closure. The cycle timeline, shown in Figure S4, is estimated from experimental measurements for wild-type *Caulobacter* strain (CB15N) grown in M2G or PYE at 28°C.^80^^,^^83^

For the case of mutant cells, where the level of CtrA∼P is either too low or too high to trigger DNA replication (Table S2), we enforce the following operations:

1. if the average [CtrA∼P] is lower than the threshold Θ at *t* = 15 min, then set *T*_ini_ = 15 min;
2. if the average [CtrA∼P] has never been lower than Θ by *t* = 300 min, then terminate the simulation at 300 min.

The transcription of some genes is regulated by CtrA∼P (see red squares with plus and minus in Figure 2.) We use Hill functions to describe transcriptional activation and inhibition by CtrA∼P as follows:

$$H_{a}\left(\text{CtrA}∼P\right)=\frac{{\left[\text{CtrA}∼P\right]}^{n}}{J_{a,\text{CtrAP}}^{n}+{\left[\text{CtrA}∼P\right]}^{n}}\text{,}$$

$$H_{i}\left(\text{CtrA}∼P\right)=\frac{J_{i,\text{CtrAP}}^{n}}{J_{i,\text{CtrAP}}^{n}+{\left[\text{CtrA}∼P\right]}^{n}}\text{.}$$

where the subscripts a and i denote activation and inhibition. *n* is the Hill exponent and *J* denotes the dissociation constant for CtrA∼P binding to the promoter.

Finally, because mRNAs diffuse slowly (∼0.03μm^2^/min)^84^ and *Caulobacter* poles are void of chromosomes,^37^ we assume that proteins are translated only in central compartments, i.e., the synthesis rates (*k*_s_) are set to 0 in polar compartments.

In total, there are 39 PDEs that describe our model of spatiotemporal regulation of the *Caulobacter* cell cycle, involving 110 parameters.

#### Multiobjective optimization

We use MATLAB’s built-in multiobjective optimization genetic algorithm (‘gamultiobj’) to estimate parameters. The 110 parameters of the model are divided into two groups: ‘fixed’ and ‘estimated’ parameters. The 69 fixed parameters include 33 parameters that have been estimated from experimental or mathematical publications, and 36 parameters that were estimated from our preliminary trials of simpler scaffolding-protein models (such as the model in Xu and Cao^44^) and tuned slightly and manually in this study. The remaining 41 parameters are chosen for parameter estimation.

Let χ∈R^41^ be the vector of estimated parameter values. We define two objective functions, *f*_1_(χ) and *f*_2_(χ). First,

$$f_{1}\left(\chi \right)=\sum_{\text{cycle}2}^{\text{cycle}3}\sum_{j}\frac{{\text{Weight}}_{j}}{n}\times \sum_{i}^{n}{\left(\frac{x_{i,j}-y_{i,j}}{\max\left(y_{j}\right)}\right)}^{2}+{\left(\max(0,|T_{\text{ini}}-25|-5\right)}^{2}),$$

where *x*_*i*,*j*_ indicates the simulated value of protein *j* at time *t*_*i*_, and *y*_*i*,*j*_ indicates the ‘scaled’ observed level of total protein *j* at time *t*_*i*_ of WT cells (see ‘quantification and statistical analysis’ for an explanation of how observed protein levels are scaled). In our model, protein *j* varies over the proteins PodJL, PodJS, and total CtrA. *n* is the number of time points for protein *j*, being 9, 9, and 8 for PodJL, PodJS, and total CtrA, respectively. Weight_*j*_ is the weight assigned to the data on protein *j*, indicating how much the discrepancy between simulated and observed levels of protein *j* contributes to the score of the objective function (Weight_PodJL_ = 80; Weight_PodJS_ = 80; Weight_totalCtrA_ = 400). We compare observed values to simulation results for the second and the third cycle in order to avoid bias in initial values. The first objective function also includes a cost of fitting the time of initiation of DNA replication (for WT cells).

The second objective function,

$$f_{2}\left(\chi \right)=\sum_{\text{cycle}2}^{\text{cycle}3}\sum_{j}{\text{Weight}}_{j}\cdot {\text{SP}}_{j}\text{,}$$

calculates a spatial fitting cost (for WT simulation) that includes penalties related to the spatial dynamics of PopZ and DivK. SP_*j*_ is the spatial penalty of protein *j*, and Weight_*j*_ denotes the contribution of SP_*j*_ to the score of the second objective function (Weight_PopZ_ = 100; Weight_DivK_ = 1). The spatial penalties are defined as:

$${\text{SP}}_{\text{PopZ}}={\left(\min\left(0,\frac{{\left[\text{PopZ}\right]}_{N}-4\cdot {\left[\text{PopZ}\right]}_{C}}{{\left[\text{PopZ}\right]}_{A}}\right)\right)}^{2}+{\left(\min\left(0,\frac{{\left[\text{PopZ}\right]}_{O}-1.5\cdot {\left[\text{PopZ}\right]}_{C}}{{\left[\text{PopZ}\right]}_{A}}\right)\right)}^{2}$$

$${\text{SP}}_{\text{DivK}}={\left(\min\left(0,\frac{{\left[\text{DivK}∼P\right]}_{N}-2\cdot {\left[\text{DivK}∼P\right]}_{C}}{{\left[\text{DivK}∼P\right]}_{A}}\right)\right)}^{2}$$

The subscripts ‘N’ and ‘O’ denote the average concentration over the cell cycle of the indicated protein in the new polar compartment and the old polar compartment. ‘C’ and ‘A’ denote the average concentration in the eight central compartments and in all ten compartments. SP_PopZ_ forces a bipolar pattern of PopZ in WT simulation and SP_DivK_ favors the transient accumulation of DivK∼P at the new pole.

Note that these two objective functions include only 1) temporal data of PodJ and CtrA, 2) spatial observations of PopZ and DivK, and 3) the initiation time of DNA replication. Other observations of WT cells and the phenotypes of all mutant strains are not considered during parameter estimation by the genetic algorithm. They are used to validate the model.

To improve the efficiency of searching the 41-dimensional parameter space, we first apply the parameter-optimization algorithm to the four-compartment model described in Methods S3. The parameter values derived from the optimized four-compartment model are used as the seed to search the parameter space in the ten-compartment model.

Each round of multiobjective optimization provides many vectors (χ’s) of estimated parameter values giving divergent values of the objective functions *f*_1_(χ) and *f*_2_(χ). Checking the vectors with relatively small values of both *f*_1_(χ) and *f*_2_(χ), we choose the parameter vector that gives the most reasonable behavior for known mutant strains. (We call this choice the parameter ‘verification’ step.) We then use the verified parameter vector as the seed for a new round of parameter optimization and verification. (During parameter optimization, the upper and lower bounds are, respectively, 1.5-fold and 0.4-fold of parameter values in the seed set.) We iterated this process many times before settling on the ‘optimum’ set of parameter values given in Table S4. All simulations reported here are based on this parameter set.

### Quantification and statistical analysis

All western blots obtained from the literature were analyzed by ImageJ to provide quantitative data for the concentration of proteins over time. Quantitative data of concentration was first normalized by $y_{i}^{\prime }=\frac{y_{i}}{\max\left(Y\right)}$, where *Y* = [*y*_1_ *y*_2_ … *y*_*i*_ … ], and then scaled to simulated results for comparison. The scaling algorithm is:

$$\underset{scale}{\arg\;\min}\sum {\left(x_{i}-scale\times y_{i}\right)}^{2}$$

where *x*_*i*_ and *y*_*i*_ are (for each observed protein time course) the simulated value and normalized quantitative data at time point *t*_*i*_.

## Data Availability

• No raw data was collected for this study. All results were produced from deterministic simulations. • All codes used for simulations and figure generation in this study can be found at https://github.com/chunruixu/Turing-pattern-model-of-scaffolding-proteins.git. • Any additional information required to reanalyze the data reported in this study is available from the lead contact upon request.

## Supplemental information

Document S1. Methods S1–S3, Tables S1–S5, and Figures S1–S7

Table S4. Estimated parameter values, related to STAR Methods
