# Supplementary material for: Turing-pattern model of scaffolding proteins that establish spatial asymmetry during the cell cycle of Caulobacter crescentus
Source: iScience. 2023 Mar 29;26(4):106513. doi: 10.1016/j.isci.2023.106513 (PMC10148035; doi:10.1016/j.isci.2023.106513)
Supplement: Document S1. Methods S1–S3, Tables S1–S5, and Figures S1–S7 [file mmc1.pdf]

iScience, Volume *26*

Supplemental information

Turing-pattern model of scaffolding proteins that establish spatial asymmetry during the cell cycle of *Caulobacter crescentus*

Chunrui Xu, John J. Tyson, and Yang Cao

Methods S1: Assumptions, related to INTRODUCTION and STAR METHODS

We summarize the assumptions made for this spatiotemporal model.

- For the scaffolding protein module (Fig. 2, blue box), we assume that:
  - 1. the periodic accumulation of scaffolding proteins at the poles is mainly driven by a Turing-pattern mechanism;
  - 2. PopZ and PodJ have slightly higher affinity with poles.
- For the DivL-CckA-CtrA module (Fig. 2, yellow box), we assume that:
  - 1. CckA has two types of binding sites: a localization site that binds to DivL or PopZ to influence the localization of CckA, and a catalytic regulatory site that interacts with DivL or DivL:DivK~P to determine the catalytic activity of CckA (kinase or phosphatase).
  - 2. PopZ binds to the unphosphorylated form of CpdR, while phosphorylation only takes place in the free form of CpdR.
- For modeling cell cycle progression, we assume that:
  - 1. when [CtrA~P] drops below a threshold, chromosome replication is initiated.
  - 2. the expression of genes that possess promoters with methylation sites is suppressed when the promoter is fully methylated.
  - 3. during the *Caulobacter* WT cell cycle, chromosome replication requires 90 min, Z-ring structure is completely closed 5 min after replication termination, and daughter cells are completely separated 25 min after Z-ring closure.

Methods S2: Partial differential equations of the spatiotemporal model, related to STAR METHODS

$$(1)\ \frac{d[\mathrm{PopZ_m}]}{dt} = k_{\mathrm{s,PopZ}} - (k_{\mathrm{d,PopZ}} + \mu) \cdot [\mathrm{PopZ_m}] + k_{\mathrm{depol,PopZ}} \cdot [\mathrm{PopZ_p}] - k_{\mathrm{dnv,PopZ}} \cdot (1 + \alpha_{\mathrm{PopZPodJ}} \cdot [\mathrm{PodJL_T}]) \cdot [\mathrm{PopZ_m}] - k_{\mathrm{aut,PopZ}} \cdot [\mathrm{PopZ_m}] \cdot [\mathrm{PopZ_p}]^2 + D_{\mathrm{PopZ_m}} \cdot \frac{\partial^2 [\mathrm{PopZ_m}]}{\partial x^2}$$

$$(2)\ \frac{d[\mathrm{PopZ_p}]}{dt} = -(k_{\mathrm{d,PopZ}} + \mu) \cdot [\mathrm{PopZ_p}] - k_{\mathrm{depol,PopZ}} \cdot [\mathrm{PopZ_p}] + k_{\mathrm{dnv,PopZ}} \cdot (1 + \alpha_{\mathrm{PopZPodJ}} \cdot [\mathrm{PodJL_T}]) \cdot [\mathrm{PopZ_m}] + k_{\mathrm{aut,PopZ}} \cdot [\mathrm{PopZ_m}] \cdot [\mathrm{PopZ_p}]^2 + D_{\mathrm{PopZ_p}} \cdot \frac{\partial^2 [\mathrm{PopZ_p}]}{\partial x^2}$$

$$(3)\ \frac{d[\mathrm{PodJL_m}]}{dt} = k_{\mathrm{s,PodJ}} \cdot \left((1-\epsilon) \cdot S_{podJ} + \epsilon\right) + k_{\mathrm{s,PodJ2}} \cdot \frac{J^4_{\mathrm{i,PodJCtrA}}}{J^4_{\mathrm{i,PodJCtrA}} + [\mathrm{CtrA{\sim}P}]^4} - (k_{\mathrm{d,PodJ1}} + k_{\mathrm{d,PodJ2}} \cdot [\mathrm{PerP}] + \mu) \cdot [\mathrm{PodJL_m}] + k_{\mathrm{depol,PodJ}} \cdot [\mathrm{PodJL_p}] - k_{\mathrm{dnv,PodJ}} \cdot [\mathrm{PodJL_m}] - \frac{k_{\mathrm{aut,PodJ}}}{1 + \alpha_{\mathrm{PodJSpmX}} \cdot [\mathrm{SpmX_T}]} \cdot [\mathrm{PodJL_m}] \cdot [\mathrm{PodJL_p}]^2 + D_{\mathrm{PodJL_m}} \cdot \frac{\partial^2 [\mathrm{PodJL_m}]}{\partial x^2}$$

$$(4)\ \frac{d[\mathrm{PodJL_p}]}{dt} = -(k_{\mathrm{d,PodJ1}} + k_{\mathrm{d,PodJ2}} \cdot [\mathrm{PerP}] + \mu) \cdot [\mathrm{PodJL_p}] - k_{\mathrm{depol,PodJ}} \cdot [\mathrm{PodJL_p}] + k_{\mathrm{dnv,PodJ}} \cdot [\mathrm{PodJL_m}] + \frac{k_{\mathrm{aut,PodJ}}}{1 + \alpha_{\mathrm{PodJSpmX}} \cdot [\mathrm{SpmX_T}]} \cdot [\mathrm{PodJL_m}] \cdot [\mathrm{PodJL_p}]^2 + D_{\mathrm{PodJL_p}} \cdot \frac{\partial^2 [\mathrm{PodJL_p}]}{\partial x^2}$$

$$(5)\ \frac{d[\mathrm{PodJS}]}{dt} = (k_{\mathrm{d,PodJ1}} + k_{\mathrm{d,PodJ2}} \cdot [\mathrm{PerP}]) \cdot ([\mathrm{PodJL_m}] + [\mathrm{PodJL_p}]) - (k_{\mathrm{d,PodJS}} + \mu) \cdot [\mathrm{PodJS}]$$

$$(6)\ \frac{d[\mathrm{SpmX_m}]}{dt} = k_{\mathrm{s,SpmX}} \cdot \frac{[\mathrm{CtrA{\sim}P}]^2}{J_{\mathrm{a,SpmXCtrA}}^2 + [\mathrm{CtrA{\sim}P}]^2} - (k_{\mathrm{d,SpmX}} + \mu) \cdot [\mathrm{SpmX_m}] + k_{\mathrm{depol,SpmX}} \cdot [\mathrm{SpmX_p}] - k_{\mathrm{dnv,SpmX}} \cdot (1 + \alpha_{\mathrm{SpmXPopZ}} \cdot [\mathrm{PopZ_T}]) \cdot [\mathrm{SpmX_m}] - k_{\mathrm{aut,SpmX}} \cdot [\mathrm{SpmX_m}] \cdot [\mathrm{SpmX_p}]^2 + D_{\mathrm{SpmX_m}} \cdot \frac{\partial^2 [\mathrm{SpmX_m}]}{\partial x^2}$$

$$(7)\ \frac{d[\mathrm{SpmX_p}]}{dt} = -(k_{\mathrm{d,SpmX}} + \mu) \cdot [\mathrm{SpmX_p}] - k_{\mathrm{depol,SpmX}} \cdot [\mathrm{SpmX_p}] + k_{\mathrm{dnv,SpmX}} \cdot (1 + \alpha_{\mathrm{SpmXPopZ}} \cdot [\mathrm{PopZ_T}]) \cdot [\mathrm{SpmX_m}] + k_{\mathrm{aut,SpmX}} \cdot [\mathrm{SpmX_m}] \cdot [\mathrm{SpmX_p}]^2 + D_{\mathrm{SpmX_p}} \cdot \frac{\partial^2 [\mathrm{SpmX_p}]}{\partial x^2}$$

$$(8)\ \frac{d[\mathrm{CtrA_u}]}{dt} = k_{\mathrm{s,CtrA1}} \cdot \left(1 - \frac{[\mathrm{CtrA{\sim}P}]}{J_{\mathrm{i,CtrACtrA}} + [\mathrm{CtrA{\sim}P}] + [\mathrm{CtrA_u}]}\right) \cdot ((1-\epsilon) \cdot S_{ctrA} + \epsilon) + k_{\mathrm{s,CtrA2}} \cdot \frac{[\mathrm{CtrA{\sim}P}]}{J_{\mathrm{a,CtrACtrA}} + [\mathrm{CtrA{\sim}P}] + [\mathrm{CtrA_u}]} - \left(k_{\mathrm{d,CtrA1}} + k_{\mathrm{d,CtrA2}} \cdot \frac{[\mathrm{CpdR_{uT}}]}{J_{\mathrm{d,CtrA}} + [\mathrm{CpdR_{uT}}]} + \mu\right) \cdot [\mathrm{CtrA_u}] - k_{\mathrm{b,CtrAuCckAkin}} \cdot ([\mathrm{CckA_{kin}}] + [\mathrm{CckA_{Z/L,kin}}]) \cdot [\mathrm{CtrA_u}] + (k_{\mathrm{ub,CtrAuCckAkin}} + k_{\mathrm{d,CckA}}) \cdot ([\mathrm{CtrA_u{:}CckA_{kin}}] + [\mathrm{CtrA_u{:}CckA_{Z/L,kin}}]) + k_{\mathrm{dephoCtrA}} \cdot ([\mathrm{CtrAP{:}CckA_{ph}}] + [\mathrm{CtrAP{:}CckA_{Z/L,ph}}]) + D_{\mathrm{CtrA}} \cdot \frac{\partial^2 [\mathrm{CtrA_u}]}{\partial x^2}$$

$$(9)\ \frac{d[\mathrm{CtrA{\sim}P}]}{dt} = -\left(k_{\mathrm{d,CtrA1}} + k_{\mathrm{d,CtrA2}} \cdot \frac{[\mathrm{CpdR_{uT}}]}{J_{\mathrm{d,CtrA}} + [\mathrm{CpdR_{uT}}]} + \mu\right) \cdot [\mathrm{CtrA{\sim}P}] - k_{\mathrm{b,CtrAPCckAph}} \cdot ([\mathrm{CckA_{ph}}] + [\mathrm{CckA_{Z/L,ph}}]) \cdot [\mathrm{CtrA{\sim}P}] + (k_{\mathrm{ub,CtrAPCckAph}} + k_{\mathrm{d,CckA}}) \cdot ([\mathrm{CtrAP{:}CckA_{ph}}] + [\mathrm{CtrAP{:}CckA_{Z/L,ph}}]) + k_{\mathrm{phoCtrA}} \cdot ([\mathrm{CtrA_u{:}CckA_{kin}}] + [\mathrm{CtrA_u{:}CckA_{Z/L,kin}}]) + D_{\mathrm{CtrA}} \cdot \frac{\partial^2 [\mathrm{CtrA{\sim}P}]}{\partial x^2}$$

$$(10)\ \frac{d[\mathrm{PleC}]}{dt} = k_{\mathrm{s,PleC}} \cdot ((1 - \epsilon_{pleC}) \cdot S_{pleC} + \epsilon) - (k_{\mathrm{d,PleC}} + \mu) \cdot [\mathrm{PleC}] - k_{\mathrm{fb,PleC}} \cdot [\mathrm{PodJL_p}] \cdot [\mathrm{PleC}] + k_{\mathrm{bf,PleC}} \cdot [\mathrm{PleC_J}] - k_{\mathrm{b,PleCDivKP}} \cdot [\mathrm{PleC}] \cdot [\mathrm{DivK{\sim}P}] + (k_{\mathrm{ub,PleCDivKP}} + k_{\mathrm{d,DivK}} + k_{\mathrm{depho,DivK}}) \cdot [\mathrm{PleC{:}DivKP}] + D_{\mathrm{PleC}} \cdot \frac{\partial^2 [\mathrm{PleC}]}{\partial x^2}$$

$$(11)\ \frac{d[\mathrm{PleC_J}]}{dt} = -\left(k_{\mathrm{d,PleC}} + \mu\right) \cdot [\mathrm{PleC_J}] + k_{\mathrm{fb,PleC}} \cdot \left[\mathrm{PodJL_p}\right] \cdot [\mathrm{PleC}] - k_{\mathrm{bf,PleC}} \cdot [\mathrm{PleC_J}] - k_{\mathrm{b,PleCDivKP}} \cdot [\mathrm{PleC_J}] \cdot [\mathrm{DivK{\sim}P}] + \left(k_{\mathrm{ub,PleCDivKP}} + k_{\mathrm{d,DivK}} + k_{\mathrm{depho,DivK}}\right) \cdot [\mathrm{PleC_J{:}DivKP}]$$

$$(12)\ \frac{d[\mathrm{PleC{:}DivKP}]}{dt} = -\left(k_{\mathrm{d,PleC}} + k_{\mathrm{d,DivK}} + k_{\mathrm{ub,PleCDivKP}} + k_{\mathrm{depho,DivK}} + k_{\mathrm{ph2kin,PleC1}} + \mu\right) \cdot [\mathrm{PleC{:}DivKP}] + k_{\mathrm{b,PleCDivKP}} \cdot [\mathrm{PleC}] \cdot [\mathrm{DivK{\sim}P}] - k_{\mathrm{fb,PleC}} \cdot \left[\mathrm{PodJL_p}\right] \cdot [\mathrm{PleC{:}DivKP}] + k_{\mathrm{bf,PleC}} \cdot [\mathrm{PleC_J}: \mathrm{DivKP}] + D_{\mathrm{PleCDivKP}} \cdot \frac{\partial^2 [\mathrm{PleC{:}DivKP}]}{\partial x^2}$$

$$(13)\ \frac{d[\mathrm{PleC_J{:}DivKP}]}{dt} = -\left(k_{\mathrm{d,PleC}} + k_{\mathrm{d,DivK}} + k_{\mathrm{ub,PleCDivKP}} + k_{\mathrm{depho,DivK}} + k_{\mathrm{ph2kin,PleC2}} + \mu\right) \cdot [\mathrm{PleC_J{:}DivKP}] + k_{\mathrm{b,PleCDivKP}} \cdot [\mathrm{PleC_J}] \cdot [\mathrm{DivK{\sim}P}] + k_{\mathrm{fb,PleC}} \cdot \left[\mathrm{PodJL_p}\right] \cdot [\mathrm{PleC{:}DivKP}] - k_{\mathrm{bf,PleC}} \cdot [\mathrm{PleC_J}: \mathrm{DivKP}]$$

$$(14)\ \frac{d[\mathrm{PleC_{kin}}]}{dt} = -\left(k_{\mathrm{d,PleC}} + \mu\right) \cdot [\mathrm{PleC_{kin}}] - k_{\mathrm{fb,PleC}} \cdot \left[\mathrm{PodJL_p}\right] \cdot [\mathrm{PleC_{kin}}] + k_{\mathrm{bf,PleC}} \cdot \left[\mathrm{PleC_{J,kin}}\right] + k_{\mathrm{ph2kin,PleC1}} \cdot [\mathrm{PleC{:}DivKP}] + D_{\mathrm{PleC}} \cdot \frac{\partial^2 [\mathrm{PleC_{kin}}]}{\partial x^2}$$

$$(15)\ \frac{d[\mathrm{PleC_{J,kin}}]}{dt} = -\left(k_{\mathrm{d,PleC}} + \mu\right) \cdot \left[\mathrm{PleC_{J,kin}}\right] + k_{\mathrm{fb,PleC}} \cdot \left[\mathrm{PodJL_p}\right] \cdot [\mathrm{PleC_{kin}}] - k_{\mathrm{bf,PleC}} \cdot \left[\mathrm{PleC_{J,kin}}\right] + +k_{\mathrm{ph2kin,PleC2}} \cdot [\mathrm{PleC_J{:}DivKP}]$$

$$(16)\ \frac{d[\mathrm{DivJ}]}{dt} = k_{\mathrm{s,DivJ}} - \left(k_{\mathrm{d,DivJ}} + \mu\right) \cdot [\mathrm{DivJ}] - k_{\mathrm{fb,DivJ}} \cdot \left[\mathrm{SpmX_p}\right] \cdot [\mathrm{DivJ}] + k_{\mathrm{bf,DivJ}} \cdot [\mathrm{DivJ_X}] - k_{\mathrm{b,DivJDivKP}} \cdot [\mathrm{DivJ}] \cdot [\mathrm{DivK{\sim}P}] + \left(k_{\mathrm{ub,DivJDivKP}} + k_{\mathrm{d,DivK}}\right) \cdot [\mathrm{DivJ{:}DivKP}] - k_{\mathrm{b,DivJDivK}} \cdot [\mathrm{DivJ}] \cdot [\mathrm{DivK}] + \left(k_{\mathrm{ub,DivJDivK}} + k_{\mathrm{d,DivK}}\right) \cdot [\mathrm{DivJ{:}DivK}] + D_{\mathrm{DivJ}} \cdot \frac{\partial^2 [\mathrm{DivJ}]}{\partial x^2}$$

$$(17)\ \frac{d[\mathrm{DivJ_X}]}{dt} = -\left(k_{\mathrm{d,DivJ}} + \mu\right) \cdot [\mathrm{DivJ_X}] + k_{\mathrm{fb,DivJ}} \cdot \left[\mathrm{SpmX_p}\right] \cdot [\mathrm{DivJ}] - k_{\mathrm{bf,DivJ}} \cdot [\mathrm{DivJ_X}] - k_{\mathrm{b,DivJDivKP}} \cdot [\mathrm{DivJ_X}] \cdot [\mathrm{DivK{\sim}P}] + \left(k_{\mathrm{ub,DivJDivKP}} + k_{\mathrm{d,DivK}}\right) \cdot [\mathrm{DivJ_X{:}DivKP}] - k_{\mathrm{b,DivJDivK}} \cdot [\mathrm{DivJ_X}] \cdot [\mathrm{DivK}] + \left(k_{\mathrm{ub,DivJDivK}} + k_{\mathrm{d,DivK}}\right) \cdot [\mathrm{DivJ_X{:}DivK}]$$

$$(18)\ \frac{d[\mathrm{DivJ{:}DivK}]}{dt} = -\left(k_{\mathrm{d,DivJ}} + k_{\mathrm{d,DivK}} + k_{\mathrm{ub,DivJDivK}} + k_{\mathrm{phoDivK,DivJ}} + \mu\right) \cdot [\mathrm{DivJ{:}DivK}] + k_{\mathrm{b,DivJDivK}} \cdot [\mathrm{DivJ}] \cdot [\mathrm{DivK}] - k_{\mathrm{fb,DivJ}} \cdot \left[\mathrm{SpmX_p}\right] \cdot [\mathrm{DivJ{:}DivK}] + k_{\mathrm{bf,DivJ}} \cdot [\mathrm{DivJ_X{:}\ DivK}] + D_{\mathrm{DivJDivK}} \cdot \frac{\partial^2[\mathrm{DivJ{:}DivK}]}{\partial x^2}$$

$$(19)\ \frac{d[\mathrm{DivJ_X{:}DivK}]}{dt} = -\left(k_{\mathrm{d,DivJ}} + k_{\mathrm{d,DivK}} + k_{\mathrm{ub,DivJDivK}} + k_{\mathrm{phoDivK,DivJ}} + \mu\right) \cdot [\mathrm{DivJ_X{:}DivK}] + k_{\mathrm{b,DivJDivK}} \cdot [\mathrm{DivJ_X}] \cdot [\mathrm{DivK}] + k_{\mathrm{fb,DivJ}} \cdot \left[\mathrm{SpmX_p}\right] \cdot [\mathrm{DivJ{:}DivK}] - k_{\mathrm{bf,DivJ}} \cdot [\mathrm{DivJ_X{:}\ DivK}]$$

$$(20)\ \frac{d[\mathrm{DivJ{:}DivKP}]}{dt} = -\left(k_{\mathrm{d,DivJ}} + k_{\mathrm{d,DivK}} + k_{\mathrm{ub,DivJDivKP}} + \mu\right) \cdot [\mathrm{DivJ{:}DivKP}] + k_{\mathrm{phoDivK,DivJ}} \cdot [\mathrm{DivJ{:}DivK}] + k_{\mathrm{b,DivJDivKP}} \cdot [\mathrm{DivJ}] \cdot [\mathrm{DivKP}] - k_{\mathrm{fb,DivJ}} \cdot \left[\mathrm{SpmX_p}\right] \cdot [\mathrm{DivJ{:}DivKP}] + k_{\mathrm{bf,DivJ}} \cdot [\mathrm{DivJ_X{:}\ DivKP}] + D_{\mathrm{DivJDivK}} \cdot \frac{\partial^2[\mathrm{DivJ{:}DivKP}]}{\partial x^2}$$

$$(21)\ \frac{d[\mathrm{DivJ_X{:}DivKP}]}{dt} = -\left(k_{\mathrm{d,DivJ}} + k_{\mathrm{d,DivK}} + k_{\mathrm{ub,DivJDivKP}} + \mu\right) \cdot [\mathrm{DivJ_X{:}DivKP}] + k_{\mathrm{phoDivK,DivJ_X}} \cdot [\mathrm{DivJ_X{:}DivK}] + k_{\mathrm{b,DivJDivKP}} \cdot [\mathrm{DivJ_X}] \cdot [\mathrm{DivKP}] + k_{\mathrm{fb,DivJ}} \cdot \left[\mathrm{SpmX_p}\right] \cdot [\mathrm{DivJ{:}DivKP}] - k_{\mathrm{bf,DivJ}} \cdot [\mathrm{DivJ_X{:}\ DivKP}]$$

$$(22)\ \frac{d[\mathrm{PerP}]}{dt} = k_{\mathrm{s,PerP}} \cdot ((1-\epsilon) \cdot S_{ctrA} + \epsilon) \cdot \frac{[\mathrm{CtrA{\sim}P}]^2}{J_{\mathrm{a,PerPCtrA}}{}^2 + [\mathrm{CtrA{\sim}P}]^2} - (k_{\mathrm{d,PerP}} + \mu) \cdot [\mathrm{PerP}] + D_{\mathrm{PerP}} \cdot \frac{\partial^2[\mathrm{PerP}]}{\partial x^2}$$

$$(23)\ \frac{d[\mathrm{DivK}]}{dt} = k_{\mathrm{s,DivK1}} + k_{\mathrm{s,DivK2}} \cdot \frac{[\mathrm{CtrA{\sim}P}]^2}{J_{\mathrm{a,DivKCtrA}}{}^2 + [\mathrm{CtrA{\sim}P}]^2} - (k_{\mathrm{d,DivK}} + \mu) \cdot [\mathrm{DivK}] - k_{\mathrm{phoDivK,PleC_{kin}}} \cdot ([\mathrm{PleC_{kin}}] + [\mathrm{PleC_{J,kin}}]) \cdot [\mathrm{DivK}] + k_{\mathrm{dephoDivK}} \cdot ([\mathrm{PleC{:}DivKP}] + [\mathrm{PleC_J{:}DivKP}]) - k_{\mathrm{b,DivJDivK}} \cdot ([\mathrm{DivJ}] + [\mathrm{DivJ_X}]) \cdot [\mathrm{DivK}] + (k_{\mathrm{ub,DivJDivK}} + k_{\mathrm{d,DivJ}}) \cdot ([\mathrm{DivJ{:}DivK}] + [\mathrm{DivJ_X{:}DivK}]) + D_{\mathrm{DivK}} \cdot \frac{\partial^2[\mathrm{DivK}]}{\partial x^2}$$

$$(24)\ \frac{d[\mathrm{DivK{\sim}P}]}{dt} = -(k_{\mathrm{d,DivK}} + \mu) \cdot [\mathrm{DivK{\sim}P}] + k_{\mathrm{phoDivK,PleC_{kin}}} \cdot ([\mathrm{PleC_{kin}}] + [\mathrm{PleC_{J,kin}}]) \cdot [\mathrm{DivK}] - k_{\mathrm{b,PleCDivKP}} \cdot ([\mathrm{PleC}] + [\mathrm{PleC_J}]) \cdot [\mathrm{DivK{\sim}P}] + (k_{\mathrm{ub,PleCDivKP}} + k_{\mathrm{d,PleC}}) \cdot ([\mathrm{PleC{:}DivKP}] + [\mathrm{PleC_J{:}DivKP}]) - k_{\mathrm{b,DivJDivKP}} \cdot ([\mathrm{DivJ}] + [\mathrm{DivJ_X}]) \cdot [\mathrm{DivK{\sim}P}] + (k_{\mathrm{ub,DivJDivKP}} + k_{\mathrm{d,DivJ}}) \cdot ([\mathrm{DivJ{:}DivKP}] + [\mathrm{DivJ_X{:}DivKP}]) - k_{\mathrm{b,DivLDivKP}} \cdot ([\mathrm{DivL}] + [\mathrm{DivL_{J/Z}}]) \cdot [\mathrm{DivK{\sim}P}] + (k_{\mathrm{ub,DivLDivKP}} + k_{\mathrm{d,DivL}}) \cdot ([\mathrm{DivL{:}DivKP}] + [\mathrm{DivL_{J/Z}{:}DivKP}]) + k_{\mathrm{ph2kin,PleC1}} \cdot [\mathrm{PleC{:}DivKP}] + k_{\mathrm{ph2kin,PleC2}} \cdot [\mathrm{PleC_J{:}DivKP}] + D_{\mathrm{DivK}} \cdot \frac{\partial^2[\mathrm{DivK{\sim}P}]}{\partial x^2}$$

$$(25)\ \frac{d[\mathrm{DivL}]}{dt} = k_{\mathrm{s,DivL}} - (k_{\mathrm{d,DivL}} + \mu) \cdot [\mathrm{DivL}] - k_{\mathrm{fb,DivL}} \cdot (\alpha_{\mathrm{DivLPopZ}} \cdot [\mathrm{PopZ_p}] + \alpha_{\mathrm{DivLPodJ}} \cdot [\mathrm{PodJ_p}]) \cdot [\mathrm{DivL}] + k_{\mathrm{bf,DivL}} \cdot [\mathrm{DivL_{J/Z}}] - k_{\mathrm{b,DivLDivKP}} \cdot [\mathrm{DivL}] \cdot [\mathrm{DivK{\sim}P}] + (k_{\mathrm{ub,DivLDivKP}} + k_{\mathrm{d,DivL}}) \cdot [\mathrm{DivL{:}DivKP}] + D_{\mathrm{DivL}} \cdot \frac{\partial^2[\mathrm{DivL}]}{\partial x^2}$$

$$(26)\ \frac{d[\mathrm{DivL_{J/Z}}]}{dt} = -(k_{\mathrm{d,DivL}} + \mu) \cdot [\mathrm{DivL_{J/Z}}] + k_{\mathrm{fb,DivL}} \cdot (\alpha_{\mathrm{DivLPopZ}} \cdot [\mathrm{PopZ_p}] + \alpha_{\mathrm{DivLPodJ}} \cdot [\mathrm{PodJ_p}]) \cdot [\mathrm{DivL}] - k_{\mathrm{bf,DivL}} \cdot [\mathrm{DivL_{J/Z}}] - k_{\mathrm{b,DivLDivKP}} \cdot [\mathrm{DivL_{J/Z}}] \cdot [\mathrm{DivK{\sim}P}] + (k_{\mathrm{ub,DivLDivKP}} + k_{\mathrm{d,DivK}}) \cdot [\mathrm{DivL_{J/Z}{:}DivKP}]$$

$$(27)\ \frac{d[\text{DivL:DivKP}]}{dt} = -\left(k_{\text{d,DivL}} + k_{\text{d,DivK}} + k_{\text{ub,DivLDivKP}} + \mu\right)\cdot[\text{DivL:DivKP}] + k_{\text{b,DivLDivKP}}\cdot[\text{DivL}]\cdot[\text{DivK\sim P}] - k_{\text{fb,DivL}}\cdot(\alpha_{\text{DivLPopZ}}\cdot\left[\text{PopZ}_{\text{p}}\right] + \alpha_{\text{DivLPodJ}}\cdot[\text{PodJ}_{\text{p}}])\cdot[\text{DivL:DivKP}] + k_{\text{bf,DivL}}\cdot[\text{DivL}_{\text{J/Z}}\text{: DivKP}] + D_{\text{DivL:DivKP}}\cdot\frac{\partial^2[\text{DivL:DivKP}]}{\partial x^2}$$

$$(28)\ \frac{d[\text{DivL}_{\text{J/Z}}\text{:DivKP}]}{dt} = -\left(k_{\text{d,DivL}} + k_{\text{d,DivK}} + k_{\text{ub,DivLDivKP}} + \mu\right)\cdot[\text{DivL}_{\text{J/Z}}\text{:DivKP}] + k_{\text{b,DivLDivKP}}\cdot[\text{DivL}_{\text{J/Z}}]\cdot[\text{DivK\sim P}] + k_{\text{fb,DivL}}\cdot\left(\alpha_{\text{DivLPopZ}}\cdot\left[\text{PopZ}_{\text{p}}\right] + \alpha_{\text{DivLPodJ}}\cdot[\text{PodJ}_{\text{p}}]\right)\cdot[\text{DivL:DivKP}] - k_{\text{bf,DivL}}\cdot[\text{DivL}_{\text{J/Z}}\text{: DivKP}]$$

$$(29)\ \frac{d[\text{CckA}_{\text{kin}}]}{dt} = k_{\text{s,CckA}} - \left(k_{\text{d,CckA}} + \mu\right)\cdot[\text{CckA}_{\text{kin}}] + k_{\text{bf,CckA}}\cdot\left[\text{CckA}_{\text{Z/L,kin}}\right] - k_{\text{fb,CckA}}\cdot\left(\alpha_{\text{CckAPopZ}}\cdot\left[\text{PopZ}_{\text{p}}\right] + \alpha_{\text{CckADivL}}\cdot[\text{DivL}_{\text{J/Z,T}}]\cdot\frac{\alpha_{\text{DivLPodJ}}\cdot[\text{PodJ}_{\text{p}}]}{\alpha_{\text{DivLPopZ}}\cdot\left[\text{PopZ}_{\text{p}}\right] + \alpha_{\text{DivLPodJ}}\cdot[\text{PodJ}_{\text{p}}]}\right)\cdot[\text{CckA}_{\text{kin}}] + \left(k_{\text{pk,CckA1}} + k_{\text{pk,CckA2}}\cdot([\text{DivL}] + [\text{DivL}_{\text{J/Z}}])\right)\cdot\left[\text{CckA}_{\text{ph}}\right] - \left(k_{\text{kp,CckA1}} + k_{\text{kp,CckA2}}\cdot([\text{DivL:DivKP}] + [\text{DivL}_{\text{J/Z}}\text{:DivKP}])\right)\cdot[\text{CckA}_{\text{kin}}] - k_{\text{b,CtrAuCckAkin}}\cdot[\text{CckA}_{\text{kin}}]\cdot[\text{CtrA}_{\text{u}}] + \left(k_{\text{d,CtrA1}} + k_{\text{d,CtrA2}}\cdot\frac{[\text{CpdR}_{\text{uT}}]}{J_{\text{d,CtrA}} + [\text{CpdR}_{\text{uT}}]} + k_{\text{ub,CtrAuCckAkin}} + k_{\text{pho,CtrA}}\right)\cdot[\text{CtrA}_{\text{u}}\text{:CckA}_{\text{kin}}] + D_{\text{CckA}}\cdot\frac{\partial^2[\text{CckA}_{\text{kin}}]}{\partial x^2}$$

$$(30)\ \frac{d[\mathrm{CckA_{Z/L,kin}}]}{dt} = -(k_{\mathrm{d,CckA}} + \mu) \cdot [\mathrm{CckA_{Z/L,kin}}] - k_{\mathrm{bf,CckA}} \cdot [\mathrm{CckA_{Z/L,kin}}] + k_{\mathrm{fb,CckA}} \cdot \left(\alpha_{\mathrm{CckAPopZ}} \cdot [\mathrm{PopZ_p}] + \alpha_{\mathrm{CckADivL}} \cdot [\mathrm{DivL_{J/Z,T}}] \cdot \frac{\alpha_{\mathrm{DivLPodJ}} \cdot [\mathrm{PodJ_p}]}{\alpha_{\mathrm{DivLPopZ}} \cdot [\mathrm{PopZ_p}] + \alpha_{\mathrm{DivLPodJ}} \cdot [\mathrm{PodJ_p}]} \right) \cdot [\mathrm{CckA_{kin}}] + \left(k_{\mathrm{pk,CckA1}} + k_{\mathrm{pk,CckA2}} \cdot ([\mathrm{DivL}] + [\mathrm{DivL_{J/Z}}]) \right) \cdot [\mathrm{CckA_{Z/L,ph}}] - \left(k_{\mathrm{kp,CckA1}} + k_{\mathrm{kp,CckA2}} \cdot ([\mathrm{DivL{:}DivKP}] + [\mathrm{DivL_{J/Z}{:}DivKP}]) \right) \cdot [\mathrm{CckA_{Z/L,kin}}] - k_{\mathrm{b,CtrAuCckAkin}} \cdot [\mathrm{CckA_{Z/L,kin}}] \cdot [\mathrm{CtrA_u}] + \left(k_{\mathrm{d,CtrA1}} + k_{\mathrm{d,CtrA2}} \cdot \frac{[\mathrm{CpdR_{uT}}]}{J_{\mathrm{d,CtrA}} + [\mathrm{CpdR_{uT}}]} + k_{\mathrm{ub,CtrAuCckAkin}} + k_{\mathrm{pho,CtrA}} \right) \cdot [\mathrm{CtrA_u{:}CckA_{Z/L,kin}}]$$

$$(31)\ \frac{d[\mathrm{CckA_{ph}}]}{dt} = -(k_{\mathrm{d,CckA}} + \mu) \cdot [\mathrm{CckA_{ph}}] + k_{\mathrm{bf,CckA}} \cdot [\mathrm{CckA_{Z/L,ph}}] - k_{\mathrm{fb,CckA}} \cdot \left(\alpha_{\mathrm{CckAPopZ}} \cdot [\mathrm{PopZ_p}] + \alpha_{\mathrm{CckADivL}} \cdot [\mathrm{DivL_{J/Z,T}}] \cdot \frac{\alpha_{\mathrm{DivLPodJ}} \cdot [\mathrm{PodJ_p}]}{\alpha_{\mathrm{DivLPopZ}} \cdot [\mathrm{PopZ_p}] + \alpha_{\mathrm{DivLPodJ}} \cdot [\mathrm{PodJ_p}]} \right) \cdot [\mathrm{CckA_{ph}}] - \left(k_{\mathrm{pk,CckA1}} + k_{\mathrm{pk,CckA2}} \cdot ([\mathrm{DivL}] + [\mathrm{DivL_{J/Z}}]) \right) \cdot [\mathrm{CckA_{ph}}] + \left(k_{\mathrm{kp,CckA1}} + k_{\mathrm{kp,CckA2}} \cdot ([\mathrm{DivL{:}DivKP}] + [\mathrm{DivL_{J/Z}{:}DivKP}]) \right) \cdot [\mathrm{CckA_{kin}}] - k_{\mathrm{b,CtrAPCckAph}} \cdot [\mathrm{CckA_{ph}}] \cdot [\mathrm{CtrA{\sim}P}] + \left(k_{\mathrm{d,CtrA1}} + k_{\mathrm{d,CtrA2}} \cdot \frac{[\mathrm{CpdR_{uT}}]}{J_{\mathrm{d,CtrA}} + [\mathrm{CpdR_{uT}}]} + k_{\mathrm{ub,CtrAPCckAph}} + k_{\mathrm{depho,CtrA}} \right) \cdot [\mathrm{CtrAP{:}CckA_{ph}}] + D_{\mathrm{CckA}} \cdot \frac{\partial^2 [\mathrm{CckA_{ph}}]}{\partial x^2}$$

$$(32)\ \frac{d[\mathrm{CckA_{Z/L,ph}}]}{dt} = -(k_{\mathrm{d,CckA}} + \mu) \cdot [\mathrm{CckA_{Z/L,ph}}] - k_{\mathrm{bf,CckA}} \cdot [\mathrm{CckA_{Z/L,ph}}] + k_{\mathrm{fb,CckA}} \cdot \left(\alpha_{\mathrm{CckAPopZ}} \cdot [\mathrm{PopZ_p}] + \alpha_{\mathrm{CckADivL}} \cdot [\mathrm{DivL_{J/Z,T}}] \cdot \frac{\alpha_{\mathrm{DivLPodJ}} \cdot [\mathrm{PodJ_p}]}{\alpha_{\mathrm{DivLPopZ}} \cdot [\mathrm{PopZ_p}] + \alpha_{\mathrm{DivLPodJ}} \cdot [\mathrm{PodJ_p}]} \right) \cdot [\mathrm{CckA_{ph}}] - \left(k_{\mathrm{pk,CckA1}} + k_{\mathrm{pk,CckA2}} \cdot ([\mathrm{DivL}] + [\mathrm{DivL_{J/Z}}]) \right) \cdot [\mathrm{CckA_{Z/L,ph}}] + \left(k_{\mathrm{kp,CckA1}} + k_{\mathrm{kp,CckA2}} \cdot ([\mathrm{DivL{:}DivKP}] + [\mathrm{DivL_{J/Z}{:}DivKP}]) \right) \cdot [\mathrm{CckA_{Z/L,kin}}] - k_{\mathrm{b,CtrAPCckAph}} \cdot [\mathrm{CckA_{ph}}] \cdot [\mathrm{CtrA{\sim}P}] + \left(k_{\mathrm{d,CtrA1}} + k_{\mathrm{d,CtrA2}} \cdot \frac{[\mathrm{CpdR_{uT}}]}{J_{\mathrm{d,CtrA}} + [\mathrm{CpdR_{uT}}]} + k_{\mathrm{ub,CtrAPCckAph}} + k_{\mathrm{depho,CtrA}} \right) \cdot [\mathrm{CtrAP{:}CckA_{Z/L,ph}}]$$

$$(33)\ \frac{d[\mathrm{CtrAP{:}CckA_{ph}}]}{dt} = -\left(k_{\mathrm{d,CckA}} + k_{\mathrm{d,CtrA1}} + k_{\mathrm{d,CtrA2}} \cdot \frac{[\mathrm{CpdR_{uT}}]}{J_{\mathrm{d,CtrA}} + [\mathrm{CpdR_{uT}}]} + k_{\mathrm{ub,CtrAPCckAph}} + k_{\mathrm{depho,CtrA}} + \mu \right) \cdot [\mathrm{CtrAP{:}CckA_{ph}}] + k_{\mathrm{b,CtrAPCckAph}} \cdot [\mathrm{CckA_{ph}}] \cdot [\mathrm{CtrA{\sim}P}] + k_{\mathrm{bf,CckA}} \cdot [\mathrm{CtrAP{:}CckA_{Z/L,ph}}] - k_{\mathrm{fb,CckA}} \cdot \left(\alpha_{\mathrm{CckAPopZ}} \cdot [\mathrm{PopZ_p}] + \alpha_{\mathrm{CckADivL}} \cdot [\mathrm{DivL_{J/Z,T}}] \cdot \frac{\alpha_{\mathrm{DivLPodJ}} \cdot [\mathrm{PodJ_p}]}{\alpha_{\mathrm{DivLPopZ}} \cdot [\mathrm{PopZ_p}] + \alpha_{\mathrm{DivLPodJ}} \cdot [\mathrm{PodJ_p}]} \right) \cdot [\mathrm{CtrAP{:}CckA_{ph}}] + D_{\mathrm{CtrACckA}} \cdot \frac{\partial^2 [\mathrm{CtrAP{:}CckA_{ph}}]}{\partial x^2}$$

$$(34)\ \frac{d\left[\mathrm{CtrAP{:}CckA_{Z/L,ph}}\right]}{dt} = -\left(k_{\mathrm{d,CckA}} + k_{\mathrm{d,CtrA1}} + k_{\mathrm{d,CtrA2}} \cdot \frac{[\mathrm{CpdR_{uT}}]}{J_{\mathrm{d,CtrA}} + [\mathrm{CpdR_{uT}}]} + k_{\mathrm{ub,CtrAPCckAph}} + k_{\mathrm{depho,CtrA}} + \mu\right) \cdot \left[\mathrm{CtrAP{:}CckA_{Z/L,ph}}\right] + k_{\mathrm{b,CtrAPCckAph}} \cdot \left[\mathrm{CckA_{Z/L,ph}}\right] \cdot [\mathrm{CtrA{\sim}P}] - k_{\mathrm{bf,CckA}} \cdot \left[\mathrm{CtrAP{:}CckA_{Z/L,ph}}\right] + k_{\mathrm{fb,CckA}} \cdot \left(\alpha_{\mathrm{CckAPopZ}} \cdot \left[\mathrm{PopZ_p}\right] + \alpha_{\mathrm{CckADivL}} \cdot \left[\mathrm{DivL_{J/Z,T}}\right] \cdot \frac{\alpha_{\mathrm{DivLPodJ}} \cdot [\mathrm{PodJ_p}]}{\alpha_{\mathrm{DivLPopZ}} \cdot \left[\mathrm{PopZ_p}\right] + \alpha_{\mathrm{DivLPodJ}} \cdot \left[\mathrm{PodJ_p}\right]}\right) \cdot \left[\mathrm{CtrAP{:}CckA_{ph}}\right]$$

$$(35)\ \frac{d\left[\mathrm{CtrA_u{:}CckA_{kin}}\right]}{dt} = -\left(k_{\mathrm{d,CckA}} + k_{\mathrm{d,CtrA1}} + k_{\mathrm{d,CtrA2}} \cdot \frac{[\mathrm{CpdR_{uT}}]}{J_{\mathrm{d,CtrA}} + [\mathrm{CpdR_{uT}}]} + k_{\mathrm{ub,CtrAuCckAkin}} + k_{\mathrm{pho,CtrA}} + \mu\right) \cdot \left[\mathrm{CtrA_u{:}CckA_{kin}}\right] + k_{\mathrm{b,CtrAuCckAkin}} \cdot [\mathrm{CckA_{kin}}] \cdot [\mathrm{CtrA_u}] + k_{\mathrm{bf,CckA}} \cdot \left[\mathrm{CtrA_u{:}CckA_{Z/L,kin}}\right] - k_{\mathrm{fb,CckA}} \cdot \left(\alpha_{\mathrm{CckAPopZ}} \cdot \left[\mathrm{PopZ_p}\right] + \alpha_{\mathrm{CckADivL}} \cdot \left[\mathrm{DivL_{J/Z,T}}\right] \cdot \frac{\alpha_{\mathrm{DivLPodJ}} \cdot [\mathrm{PodJ_p}]}{\alpha_{\mathrm{DivLPopZ}} \cdot \left[\mathrm{PopZ_p}\right] + \alpha_{\mathrm{DivLPodJ}} \cdot \left[\mathrm{PodJ_p}\right]}\right) \cdot \left[\mathrm{CtrA_u{:}CckA_{kin}}\right] + D_{\mathrm{CtrACckA}} \cdot \frac{\partial^2\left[\mathrm{CtrA_u{:}CckA_{kin}}\right]}{\partial x^2}$$

$$(36)\ \frac{d[\text{CtrA}_\text{u}\text{:CckA}_\text{Z,kin}]}{dt} = -\left(k_\text{d,CckA} + k_\text{d,CtrA1} + k_\text{d,CtrA2}\cdot\frac{[\text{CpdR}_\text{uT}]}{J_\text{d,CtrA} + [\text{CpdR}_\text{uT}]} + k_\text{ub,CtrAuCckAkin} + k_\text{pho,CtrA} + \mu\right) \cdot \left[\text{CtrA}_\text{u}\text{:CckA}_\text{Z,kin}\right] + k_\text{b,CtrAuCckAkin}\cdot\left[\text{CckA}_\text{Z,kin}\right]\cdot[\text{CtrA}_\text{u}] - k_\text{bf,CckA} \cdot\left[\text{CtrA}_\text{u}\text{:CckA}_\text{Z/L,kin}\right] + k_\text{fb,CckA} \cdot\left(\alpha_\text{CckAPopZ}\cdot\left[\text{PopZ}_\text{p}\right] + \alpha_\text{CckADivL}\cdot\left[\text{DivL}_\text{J/Z,T}\right] \cdot\frac{\alpha_\text{DivLPodJ}\cdot[\text{PodJ}_\text{p}]}{\alpha_\text{DivLPopZ}\cdot\left[\text{PopZ}_\text{p}\right] + \alpha_\text{DivLPodJ}\cdot[\text{PodJ}_\text{p}]}\right)\cdot\left[\text{CtrA}_\text{u}\text{:CckA}_\text{kin}\right]$$

$$(37)\ \frac{d[\text{CpdR}]}{dt} = k_\text{s,CpdR}\cdot\frac{[\text{CtrA\textasciitilde P}]^2}{{J_\text{a,CpdRCtrA}}^2 + [\text{CtrA\textasciitilde P}]^2} - (k_\text{d,CpdR} + \mu)\cdot[\text{CpdR}] - k_\text{fb,CpdR}\cdot\left[\text{PopZ}_\text{p}\right] \cdot[\text{CpdR}] + k_\text{bf,CpdR}\cdot\left[\text{CpdR}_\text{Z}\right] - k_\text{phoCpdR}\cdot\left([\text{CckA}_\text{kin}] + \left[\text{CckA}_\text{Z,kin}\right]\right)\cdot[\text{CpdR}] + k_\text{dephoCpdR}\cdot\left(\left[\text{CckA}_\text{ph}\right] + \left[\text{CckA}_\text{Z,ph}\right]\right)\cdot[\text{CpdR}\sim\text{P}] + D_\text{CpdR}\cdot\frac{\partial^2[\text{CpdR}]}{\partial x^2}$$

$$(38)\ \frac{d[\text{CpdR}_\text{Z}]}{dt} = -(k_\text{d,CpdR} + \mu)\cdot\left[\text{CpdR}_\text{Z}\right] + k_\text{fb,CpdR}\cdot\left[\text{PopZ}_\text{p}\right]\cdot[\text{CpdR}] - k_\text{bf,CpdR}\cdot\left[\text{CpdR}_\text{Z}\right]$$

1. Xu, C. and Cao, Y. A spatiotemporal model of polarity and spatial gradient establishment in *Caulobacter crescentus*. in Proceedings of the 12th ACM Conference on Bioinformatics, Computational Biology, and Health Informatics. 2021.

$$(39)\ \frac{d[\text{CpdR\textasciitilde P}]}{dt} = -(k_\text{d,CpdR} + \mu)\cdot[\text{CpdR\textasciitilde P}] + k_\text{phoCpdR}\cdot\left([\text{CckA}_\text{kin}] + \left[\text{CckA}_\text{Z,kin}\right]\right)\cdot[\text{CpdR}] - k_\text{dephoCpdR}\cdot\left(\left[\text{CckA}_\text{ph}\right] + \left[\text{CckA}_\text{Z,ph}\right]\right)\cdot[\text{CpdR}\sim\text{P}] + D_\text{CpdR}\cdot\frac{\partial^2[\text{CpdR\textasciitilde P}]}{\partial x^2}$$

Methods S3: The four-compartment model, related to STAR Methods

In the four-compartment model, the total cell length (L) is divided into four compartments, with two poles of length $0.2L$ each and two central compartments of length $0.3L$ each. Diffusion across the boundary between neighboring compartments causes compensatory changes in the concentrations of the diffusing species within each compartment. Hence, we describe the reaction and diffusion of proteins in a four-compartment model by a set of four ODEs for $C_i(t)$, the protein concentration in compartment i at time t [S1]:

$$\begin{cases} \frac{dC_1}{dt} = \text{CRR} + \frac{4D(C_2 - C_1)}{(l_1 + l_2)^2} \\ \frac{dC_i}{dt} = \text{CRR} + \frac{4D(C_{i+1} - C_i)}{(l_{i+1} + l_i)^2} + \frac{4D(C_{i-1} - C_i)}{(l_{i-1} + l_i)^2}, i = 2,3 \\ \frac{dC_{10}}{dt} = \text{CRR} + \frac{4D(C_3 - C_4)}{(l_3 + l_4)^2} \end{cases}$$

where l_i indicates the length of compartment i.

We show the WT cell simulation of the four-compartment model in Fig. S7.

Table S1: Predictions provided by the spatiotemporal model, related to INTRODUCTION and Fig. 6.

Predictions	Cell types
higher polar affinity for PodJ and PopZ	required by Δ*podJ* and Δ*spmX* simulations, and applied for all cell types.
distributions of SpmX and DivJ	Δ*podJ* (Fig. 6(b)).
distribution of PopZ	Δ*spmX* (Fig. 6(d) and Fig. 6(e)).
DivK~P spatial distribution	'delocalized PleC' and 'delocalized DivL' (Fig. 8(g) and Fig. 8(h));<br>Δ*podJ*, Δ*popZ*, and Δ*spmX* (Fig. S3).
CtrA~P spatial distribution	'delocalized PleC' and 'delocalized DivL' (Fig. 8(g) and Fig. 8(h));<br>Δ*podJ*, Δ*popZ*, and Δ*spmX* (Fig. S3);<br>Δ*divJ*, *divJ-H338A*, Δ*pleC*, *pleC-H610A*, *pleC-F778L* (Fig. 8).

Table S2: Events and switches of parameters, related to Results and STAR METHODS.

Event description	Condition	Change at the event
replication initiation	average [CtrA~P] drops below Θ	$T_{\mathrm{ini}} = t$
replication fork passes *ctrA*	$t > T_{\mathrm{ini}}$ + 33.3	S_{ctrA}=1
replication fork passes *pleC*	$t > T_{\mathrm{ini}}$ + 58.5	S_{pleC} =1
replication fork passes *perP*	$t > T_{\mathrm{ini}}$ + 66.6	S_{perP} =1
replication fork passes *podJ*	$t > T_{\mathrm{ini}}$ + 78.3	S_{podJ} =1
replication termination	$t > T_{\mathrm{term}}$	All '*S*'s are switched to 0

Table S3: Parameter changes for mutant simulations, related to Results and STAR METHODS

Mutant	Setting	Mutant	Setting
Δ*podJ*	$k_{\mathrm{s,PodJ}} = 0$, $k_{\mathrm{s,PodJ2}} = 0$	$\mathrm{PodJ}^{\mathrm{op1}}$	$k_{\mathrm{s,PodJ}}$ = 1.2-fold of WT $k_{\mathrm{s,PodJ}}$,<br>$k_{\mathrm{s,PodJ2}}$ = 1.2-fold of WT $k_{\mathrm{s,PodJ2}}$
$\mathrm{PodJ}^{\mathrm{op2}}$	$k_{\mathrm{s,PodJ}}$ = 12-fold of WT $k_{\mathrm{s,PodJ}}$,	Δ*popZ*	$k_{\mathrm{s,PopZ}} = 0$

	$k_{s,PodJ2}$ = 12-fold of WT $k_{s,PodJ2}$		
PopZ^{op}	$k_{s,PopZ}$ = 10-fold of WT $k_{s,PopZ}$	Δ*spmX*	$k_{s,SpmX}$ = 0
Δ*divJ*	$k_{s,divJ}$ = 0	*DivJ-H336A*	$k_{phoDivK,DivJ}$ = 0, $k_{phoDivK,DivJX}$ = 0
Δ*pleC*	$k_{s,PleC}$ = 0	*PleC-F778L*	$k_{phoDivK,PleCkin}$ =0.01-fold of WT $k_{phoDivK,PleCkin}$
delocalized PleC	$k_{fb,PleC}$ = 0	*PleC-H610A*	$k_{dephoDivK}$ = 0, $k_{phoDivK,PleCkin}$ = 0
delocalized DivL	$\alpha_{DivLPodJ}$ = 0		

Table S5: Dynamics and functions of individual species in model, related to STAR METHODS

Species	Key Dynamics	Key Functions
CtrA~P	After Z-ring closure, CtrA~P remains high in the swarmer compartment.	A high level of CtrA~P (swarmer part) inhibits DNA replication; a low level of CtrA~P (stalk part) allows DNA replication; CtrA~P regulates the expression level of *spmX*, *podJ*, *perP*, *divK*, and *ctrA*.
CtrAu	After Z-ring closure, unphosphorated CtrA is phosphorylated in the swarmer part and degraded in the stalk part.	
CckA	CckA turns from phosphatase to kinase at the new pole during the predivisional stage.	CckA phosphatase and kinase are responsible for dephosphorylating and phosphorylating CtrA and CpdR, respectively.
CpdR	The total CpdR remains at a high level; after Z-ring closure, the unphosphorylated form is high in the stalk part and the phosphorylated form is high in the swarmer part.	Unphosphorylated CpdR is responsible for the degradation of CtrA (both phosphorylated and unphosphorylated forms), thus allowing DNA replication in the nascent stalked cell.
DivK~P	DivK~P maintains a high level at the old pole and temporarily accumulates at the new pole in the predivisional stage.	DivK~P binds to DivL, DivJ, and PleC.
DivL	DivL is mainly localized at the new pole in the predivisional stage.	Free DivL stimulates the kinase activity of CckA.
DivL:DivK~P Complex	The complex forms where both DivL and DivK~P are at relatively high levels.	DivL:DivK~P stimulates the phosphatase activity of CckA, which negatively regulates both CtrA level and activity, and consequently influences the

		initiation of DNA replication.
DivJ	DivJ is co-localized with SpmX at the old pole.	DivJ phosphorylates DivK and competes with DivL and PleC to bind to DivK~P.
PleC	PleC is co-localized with PodJ at the new pole.	By dephosphorylating DivK~P, PleC stimulates the kinase activity of CckA, thereby increasing CtrA~P level.
PopZ	PopZ is persistently localized at the old pole and accumulates at the new pole due to a Turing instability and help from PodJ.	PopZ directly regulates the localization of several species, such as CckA, CpdR, DivL, SpmX, and indirectly affects the localization of DivJ.
PodJL	PodJL is localized at the new pole due to SpmX's inhibition and gets truncated to PodJS by PerP in the late predivisional stage.	PodJL recruits PleC and DivL to the new pole.
PodJS	PodJS accumulates at the new pole in the late predivisional stage and remains at this until it is degraded.	
SpmX	SpmX is recruited by PopZ and accumulates at the old pole.	SpmX recruits DivJ at the old pole.

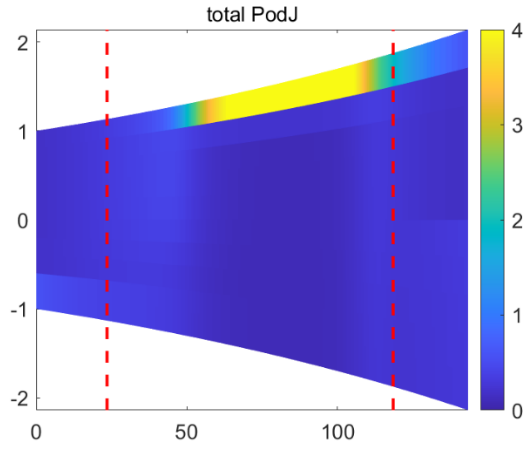

Figure S1: The spatial dynamics of total PodJ (PodJL + PodJS) in WT simulation, related to Fig. 3. and RESULTS AND DISCUSSION.

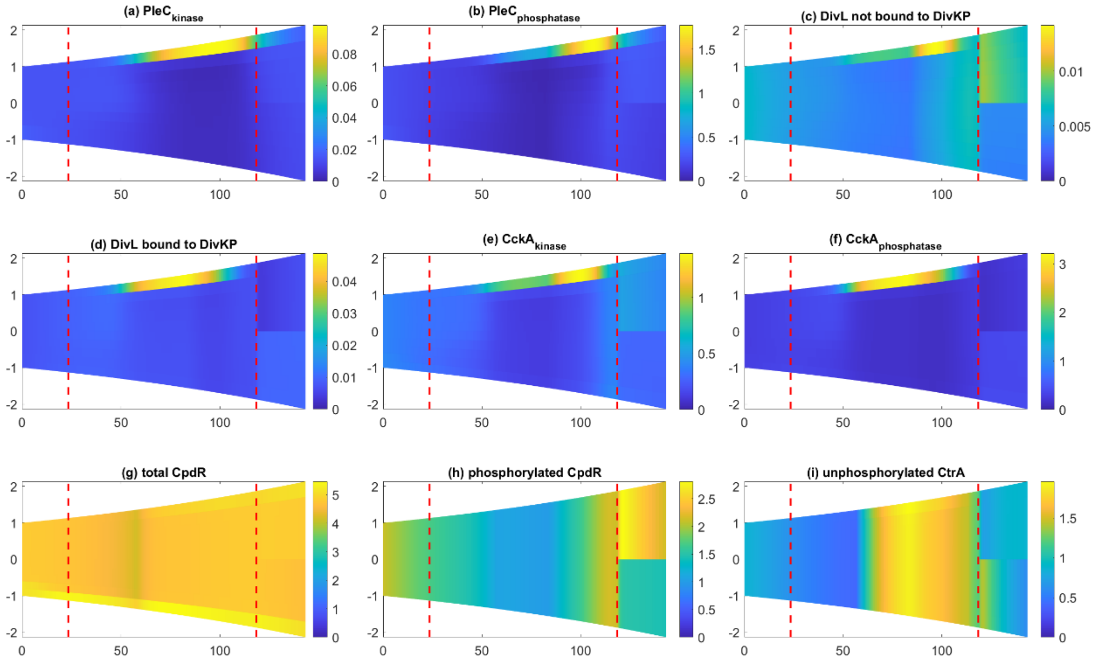

Figure S2: The spatial dynamics of PleC kinase and phosphatase, DivL unbound to DivK~P, DivL:DivK~P complex, CckA kinase and phosphatase, total CpdR, CpdR~P, and unphosphorylated CtrA in WT simulation, related to Fig. 4. and RESULTS AND DISCUSSION.

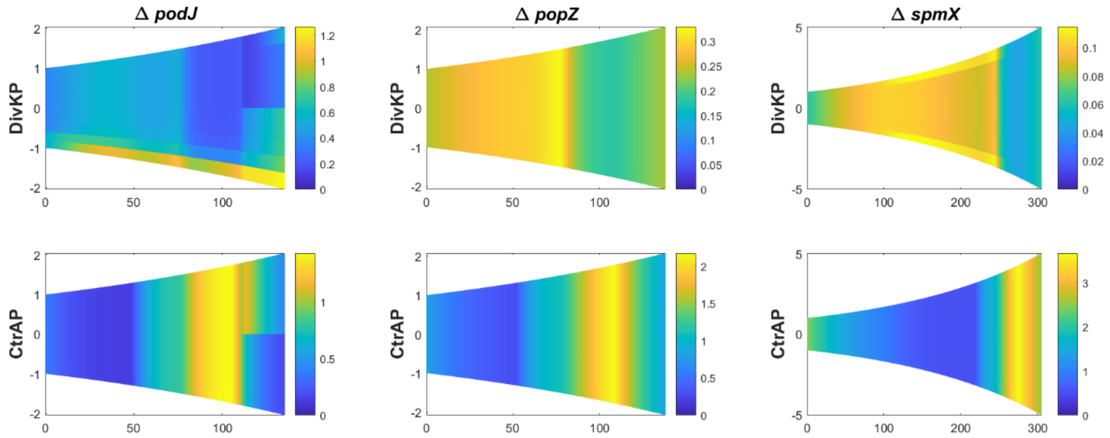

Figure S3: DivK~P and CtrA~P dynamics in the simulation of Δ*podJ*, Δ*popZ*, and Δ*spmX*, related to Fig. 6. and RESULTS AND DISCUSSION.

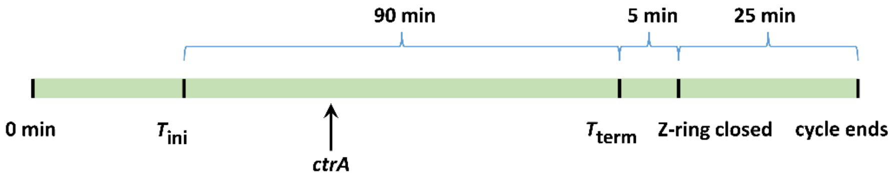

Figure S4: Schematic cell-cycle timeline for a wild-type cell, related to STAR METHODS. 0 min indicates the time when a swarmer cell is separated completely from its sibling. DNA replication initiates (T_{ini}) when [CtrA~P] drops below the threshold Θ. DNA replication needs 90 min, so the time of termination is $T_{term}=T_{ini}+90$ min. Z-ring closure is completed 5 min later, and 25 min after that the nascent swarmer cell separates from the stalked cell. *ctrA* with an arrow indicates the time, T_{ini} +33.3 min, when the replication fork passes the *ctrA* locus.

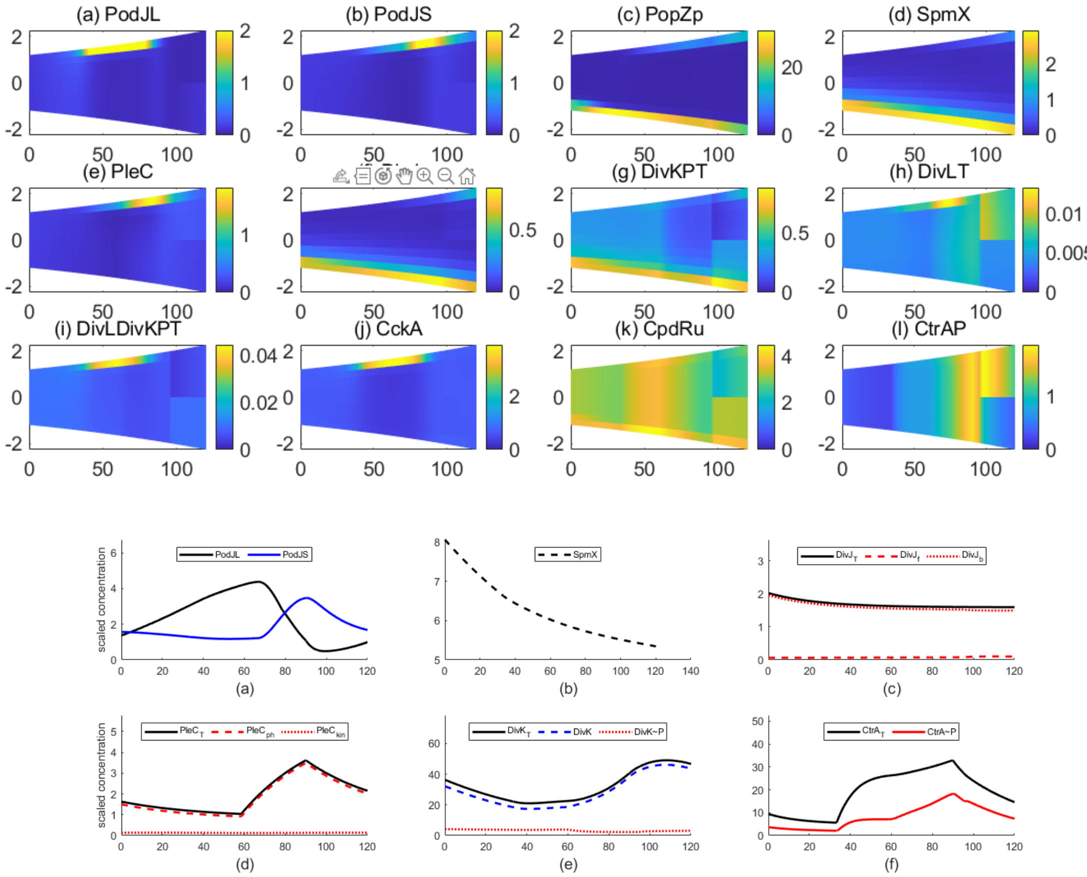

Figure S5: WT simulation of the ten-compartment model for the stalked cell cycle, related to STAR METHODS. The upper sub-figures (a)-(l) are simulated spatial dynamics of proteins or complex. The lower sub-figures (a)-(f) are simulated temporal dynamics of proteins.

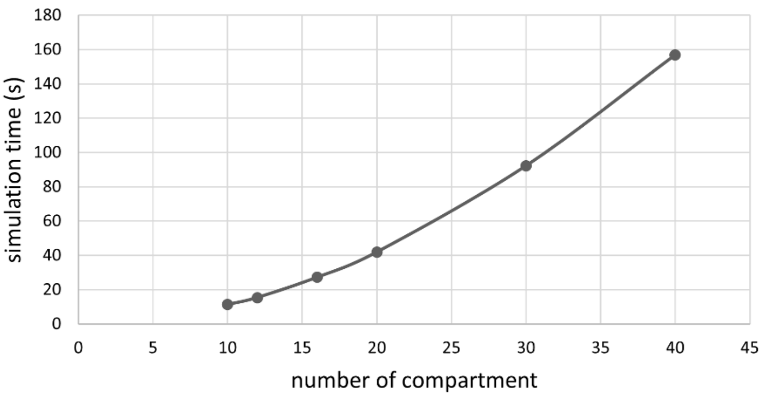

Figure S6: Model simulation time increases quadratically with N = number of compartments, related to STAR METHODS. The curve $y = 0.097x^2 + 2.216$ fits the timing data (dots) perfectly.

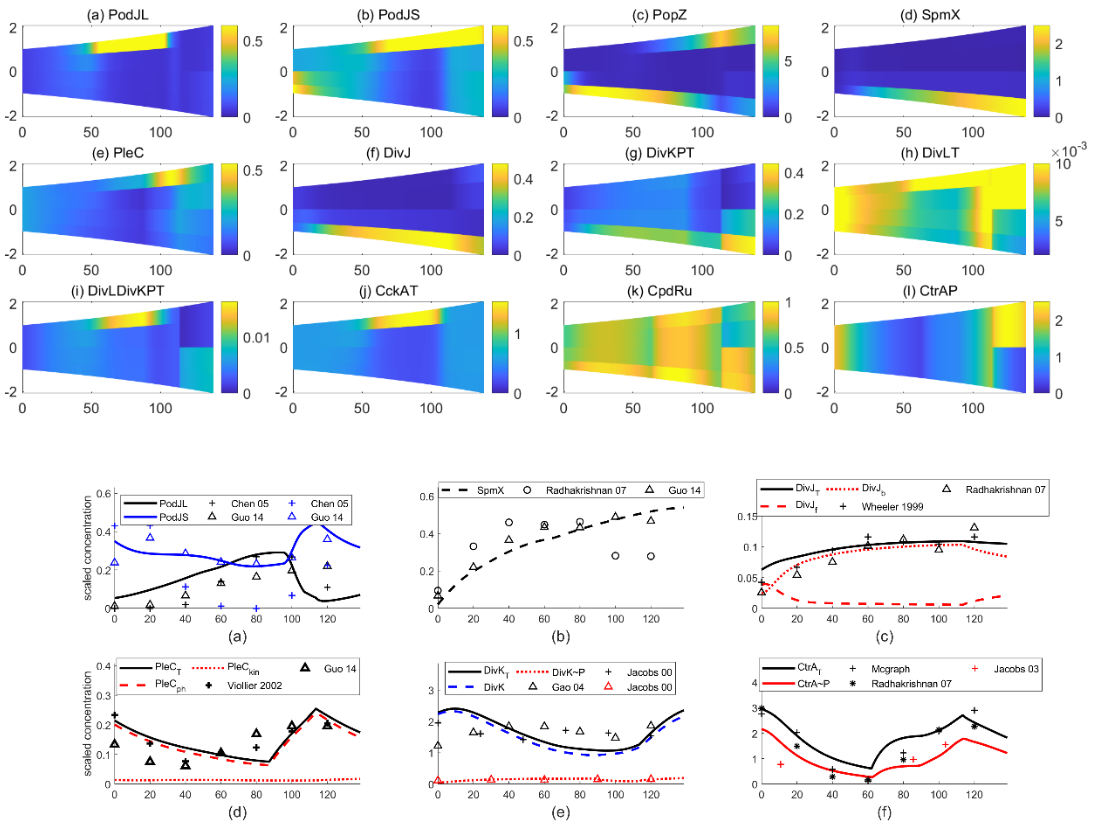

Figure S7: WT simulation of the four-compartment model, related to STAR METHODS. The upper sub-figures (a)-(l) are simulated spatial dynamics of proteins or complex. The lower sub-figures (a)-(f) are simulated temporal dynamics of proteins.

References

S1. Xu, C. and Cao, Y. (2021). A spatiotemporal model of polarity and spatial gradient establishment in *Caulobacter crescentus*. in Proceedings of the 12th ACM Conference on Bioinformatics, Computational Biology, and Health Informatics.
